# Pt@ZnCo_2_O_4_ Microspheres as Peroxidase Mimics: Enhanced Catalytic Activity and Application for L-Cysteine Detection

**DOI:** 10.3390/molecules30010187

**Published:** 2025-01-05

**Authors:** Shuqi Liao, Weisen Deng, Feng Yang, Jutao Zhou, Ling Wu, Donghong Yu, Zhong Cao

**Affiliations:** 1 Hunan Provincial Key Laboratory of Materials Protection for Electric Power and Transportation & Hunan Provincial Key Laboratory of Cytochemistry, School of Chemistry and Chemical Engineering, Changsha University of Science and Technology, Changsha 410114, China; liaoshuqi@stu.csust.edu.cn (S.L.); senweideng@163.com (W.D.); yangf2004@stu.csust.edu.cn (F.Y.); zhoujutao@stu.csust.edu.cn (J.Z.); wuling@csust.edu.cn (L.W.); 2 Department of Chemistry and Bioscience, Aalborg University, DK-9220 Aalborg, Denmark; yu@bio.aau.dk

**Keywords:** artificial mimetic enzyme, Pt@ZnCo_2_O_4_ microspheres, peroxidase-like enzymes, L-cysteine, colorimetric detection

## Abstract

Compared to natural enzymes, the development of efficient artificial simulated enzymes, such as those based on bimetallic materials with high catalytic activity and good stability, is an important way until now. Herein, we employed ZnCo_2_O_4_ microspheres as carriers to synthesize Pt-doped composites with different amounts using a one-pot method. The morphology and structure of the synthesized materials were characterized using XRD, SEM, BET, FT-IR, XPS, and Zeta potential techniques. It was found that Pt^0^ adhered well to the surface of ZnCo_2_O_4_ microspheres, with a 12.5% Pt doped ratio exhibiting abundant oxygen vacancies, excellent substrate affinity, and high peroxidase-like activity. Using fluorescent probes and electrochemical methods, the peroxidase-like catalytic mechanism has been explored that Pt@ZnCo_2_O_4_ microspheres can accelerate the electron transfer between H_2_O_2_ and 3,3′,5,5′-tetramethylbenzidine (TMB). Based on the optimal loading ratio of 12.5% of Pt@ZnCo_2_O_4_, a colorimetric sensor for visual detection of L-cysteine (L-Cys) was constructed, exhibiting a wide linear range of 0.1~50 µM and a low detection limit of 0.0163 µM. The sensor possesses good selectivity, reusability, and usage stability, which can be well applied to the determination of L-Cys in health product capsules with recovery rates of 96.9%~103.7% and RSD of 1.07%~6.50%. This work broadens the application prospects of spinel materials such as ZnCo_2_O_4_ in the field of biological analysis and also provides inspiration for the development of new artificial simulated enzymes.

## 1. Introduction

ZnCo_2_O_4_ belongs to an AB_2_O_4_-type spinel material, which has an isometric cubic spinel structure. The functional properties of ZnCo_2_O_4_ can be adjusted by varying the oxygen content in the composition [1,2]. Due to its excellent optical, electrical, and magnetic properties, as well as outstanding chemical and structural stability, spinel ZnCo_2_O_4_ has attracted increased interest of researchers and has been extensively applied in fields such as electrocatalysis [3], photocatalysis [4], energy storage [5], gas sensing [6,7], and electrochemical sensors [8]. While the remarkable optoelectronic properties and strong structural stability of spinel ZnCo_2_O_4_ have garnered significant attention, there were relatively few reports on its potential intrinsic enzymatic activity [9,10]. Moreover, the excellent chemical properties and designability of ZnCo_2_O_4_ may provide a promising platform for the development of novel artificial mimetic enzymes whose catalytic activity can be enhanced through various strategies [11]. Currently, introducing oxygen vacancies into metal oxides is recognized as an effective method for improving catalytic activity [12]. The approaches for introducing oxygen vacancies in metal oxides can be broadly categorized into two main strategies: one involves pyrolysis of metal–organic frameworks (MOFs) to prepare oxygen vacancy-rich metal oxides, while the other increases oxygen vacancies through metal doping. In comparison, the metal doping method stands out due to its simplicity and straightforward processing. This method not only enhances the oxygen vacancies in metal materials to provide more active sites but also addresses the issue of reduced catalytic activity resulting from the agglomeration of metal materials [13]. Furthermore, platinum, as a precious metal, exhibits good biocompatibility and readily provides abundant oxygen vacancies, thereby enhancing the adsorption of substrate molecules by mimetic enzymes and improving their catalytic activity [14]. Their combination and synergistic effect will help improve the catalytic performance of the materials, being a significant trend in the development of novel optoelectronic active materials for the detection of biomarker molecules.

As a conditional and glucogenic amino acid in the human body, L-cysteine (L-Cys) is involved in multiple essential physiological processes, such as protein folding, catalysis of cysteine proteases, regulation of oxidative stress and metabolic levels, and participation in physiological signal transduction [15]. Therefore, monitoring L-Cys levels is crucial for clinical diagnosis and understanding necessary physiological and pathological processes [16]. Currently, the main methods for detecting cysteine include high-performance liquid chromatography (HPLC) [17], gas chromatography (GC) [18], fluorescence spectroscopy [19,20,21], capillary electrophoresis [22], resonance light scattering techniques [23], and electrochemical detection [24,25,26]. However, most of those methods were limited by the bulky and expensive instruments, with the complexity of the operation, which cannot achieve rapid and convenient detection. In recent years, due to the advantages of simplicity, low cost, and quick speed, colorimetric methods have provided a cheap and efficient alternative for the detection of biomolecules such as amino acids [27,28,29]. One principle of colorimetric detection of L-Cys involves enzyme-catalyzed decomposition of hydrogen peroxide (H_2_O_2_) into reactive oxygen species (ROS), which subsequently oxidize an organic chromogenic agent to form a coupling product. This coupled product exhibits light absorption at a specific wavelength, and the addition of L-Cys to the system inhibits the coupling of the organic chromogenic agent, thereby reflecting the level of L-Cys through a decrease in absorbance [30]. Compared to natural enzymes, artificial mimetic enzymes, particularly those based on bimetallic materials, have attracted widespread attention due to their advantages of simplicity in preparation, low cost, good stability, and high catalytic efficiency [31]. Therefore, an efficient and convenient colorimetric detection platform for L-Cys may be developed by utilizing the enhanced mimetic enzyme performance based on metal-doped ZnCo_2_O_4_ materials.

**Scheme 1:**
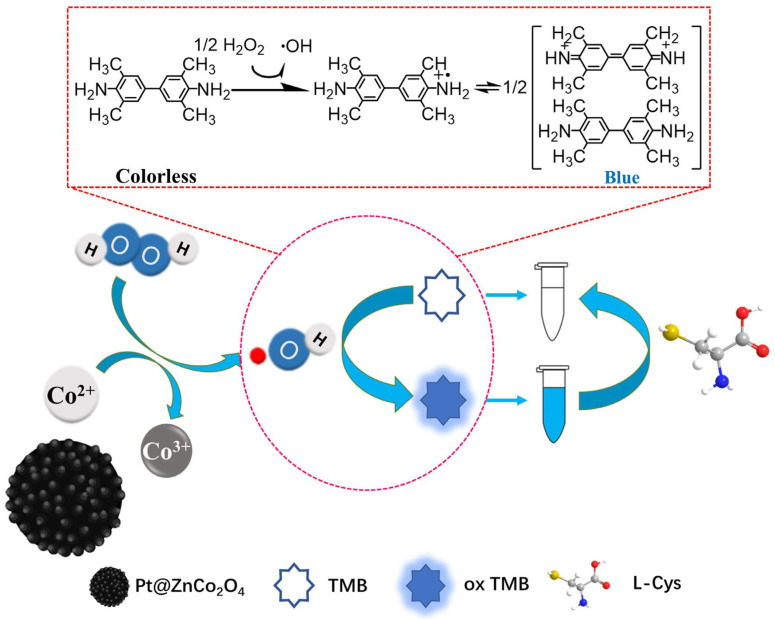
Schematic diagram for detection of L-Cys using a colorimetric sensor based on Pt@ZnCo_2_O_4_ microspheres.

In this study, the platinum-doped ZnCo_2_O_4_ microspheres (Pt@ZnCo_2_O_4_ MSs) were synthesized with varying amounts of Pt added by using a one-pot method. Compared to pure spinel structural ZnCo_2_O_4_ MSs, Pt@ZnCo_2_O_4_ MSs exhibited significantly enhanced peroxidase-like activity. The incorporation of Pt^0^ increases the oxygen vacancies in the ZnCo_2_O_4_ MSs, facilitating electron transfer between the material and substrate, thereby greatly enhancing the catalytic activity of the ZnCo_2_O_4_ MSs. The optimal Pt loading levels of 12.5% in Pt@ZnCo_2_O_4_ (atomic ratio) were found to show the highest enzymatic activity, and the catalytic mechanism of H_2_O_2_ follows the classical Fenton reaction. Based on this, a colorimetric sensor for detecting L-Cys was built (Scheme 1), achieving a detection limit of 0.0163 μM (S/N = 3). Additionally, the sensor can be successfully applied to the detection of L-Cys content in dietary supplement capsules, which is in agreement with that by using HPLC, providing a novel approach for the development of artificial mimetic enzymes as alternatives to natural enzymes.

## 2. Results and Discussion

### 2.1. Characterization of Materials

**Figure 1:**
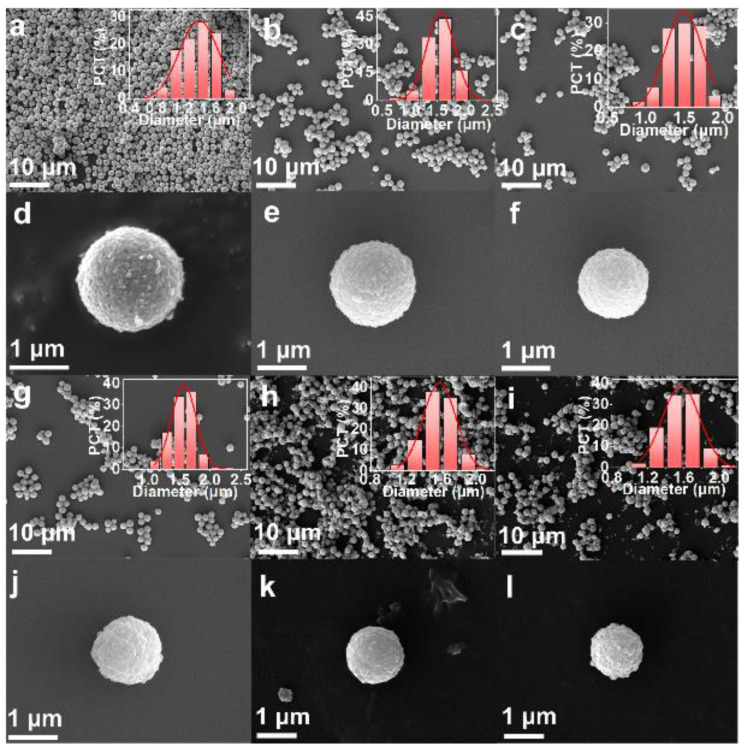
SEM images and particle size distribution charts of Pt@ZnCo_2_O_4_ microspheres with different Pt loading concentrations: (**a**) ZnCo_2_O_4_, (**b**) 4.39% Pt@ZnCo_2_O_4_, (**c**) 6.58% Pt@ZnCo_2_O_4_, (**g**) 8.31% Pt@ZnCo_2_O_4_, (**h**) 10.4% Pt@ZnCo_2_O_4_, and (**i**) 12.5% Pt@ZnCo_2_O_4_. Corresponding high-magnification SEM images: (**d**) ZnCo_2_O_4_, (**e**) 4.39% Pt@ZnCo_2_O_4_, (**f**) 6.58% Pt@ZnCo_2_O_4_, (**j**) 8.31% Pt@ZnCo_2_O_4_, (**k**) 10.4% Pt@ZnCo_2_O_4_, and (**l**) 12.5% Pt@ZnCo_2_O_4_.

Zn-Co carbonate precursors were prepared via a co-precipitation method, and Pt@ZnCo_2_O_4_ MSs with varying Pt loading concentrations were synthesized through an in situ reduction in K_2_PtCl_4_ aqueous solution based on ZnCo_2_O_4_ MSs. The morphologies and particle sizes of ZnCo_2_O_4_ and Pt@ZnCo_2_O_4_ microspheres with different Pt loading concentrations were then investigated by using a scanning electron microscopy (SEM) and laser particle size analyzer. As shown in Figure 1a, the Pt-free ZnCo_2_O_4_ exhibits uniformly dispersed porous microspheres. The particle size distribution, shown in an inset of Figure 1a, reveals that the microspheres have a diameter ranging from 700 to 1800 nm, with a peak around 1400 nm. The corresponding high-magnification SEM image (Figure 1d) clearly shows that the microsphere is composed of small aggregated particles with sizes ranging from 50 to 80 nm (Figure S1), and its surface exhibits noticeable porosity. The N₂ adsorption–desorption isotherm and pore size distribution indicate that ZnCo_2_O_4_ MSs possess both mesoporous and macroporous structures, with the main pore size around 50 nm (Figure S2). Figure 1b,c,g–i show the SEM images of Pt@ZnCo_2_O_4_ MSs with Pt loading concentrations of 4.39%, 6.58%, 8.31%, 10.4%, and 12.5%, respectively. It can be seen that the Pt loading has no effect on the overall morphology of the microspheres, which remains uniformly spherical.

However, their particle size distributions (insets of Figure 1a–i) show that the average diameters of the Pt@ZnCo_2_O_4_ MSs are increased a little bigger than that of ZnCo_2_O_4_ MSs with increased Pt concentration, with a peak around from 1400 nm to 1600 nm. In comparison with ZnCo_2_O_4_, the 4.39% and 6.58% Pt@ZnCo_2_O_4_ MSs exhibit fewer particles on their surfaces (Figure 1b,c) and less porosity. This may be due to Pt deposition on the surface of the ZnCo_2_O_4_ MSs, filling the pores formed by the aggregation of the small particles and making the surface smooth. With the Pt loading concentration increased, the 8.31%, 10.4%, and 12.5% Pt@ZnCo_2_O_4_ MSs show a gradual increase in particle size (Figure 1g–i) and surface roughness (Figure 1j–l), indicating that the control by adjusting the loading amount of Pt can modulate the specific surface area and active sites of Pt@ZnCo_2_O_4_, providing a foundation for sensing and catalytic research.

**Figure 2:**
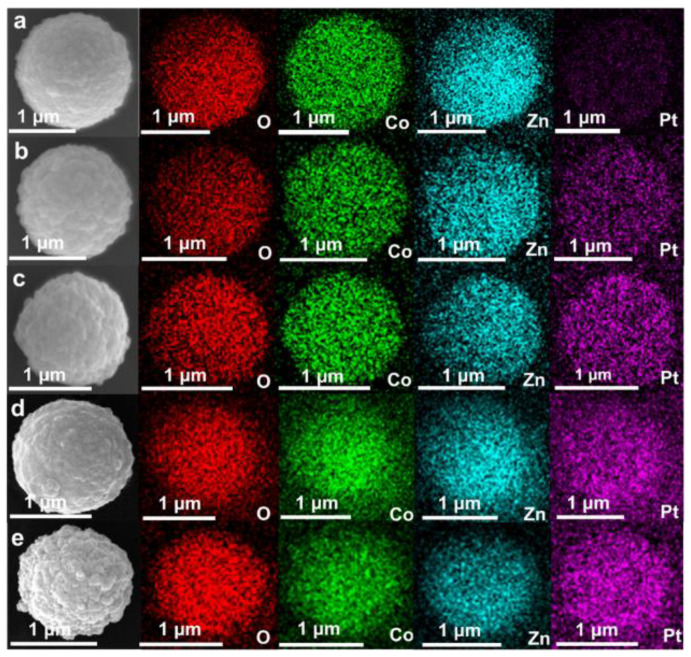
Elemental distribution maps of O, Co, Zn, and Pt for (**a**) 4.39% Pt@ZnCo_2_O_4_, (**b**) 6.58% Pt@ZnCo_2_O_4_, (**c**) 8.31% Pt@ZnCo_2_O_4_, (**d**) 10.4% Pt@ZnCo_2_O_4_, and (**e**) 12.5% Pt@ZnCo_2_O_4_.

In addition to the morphological comparison, the elemental analysis of Pt@ZnCo_2_O_4_ MSs with different Pt loading concentrations was also performed. Figure 2 presents high-resolution elemental distribution maps for different Pt concentrations (4.39%, 6.58%, 8.31%, 10.4%, and 12.5%) doped ZnCo_2_O_4_ microspheres, indicating that the content of O, Co, and Zn elements in the five materials remains almost unchanged, but the content of Pt element is gradually increased with the increasing ratio doped. It can be seen that K_2_PtCl_4_ has been successfully reduced in situ on the surface of ZnCo_2_O_4_ MSs.

**Figure 3:**
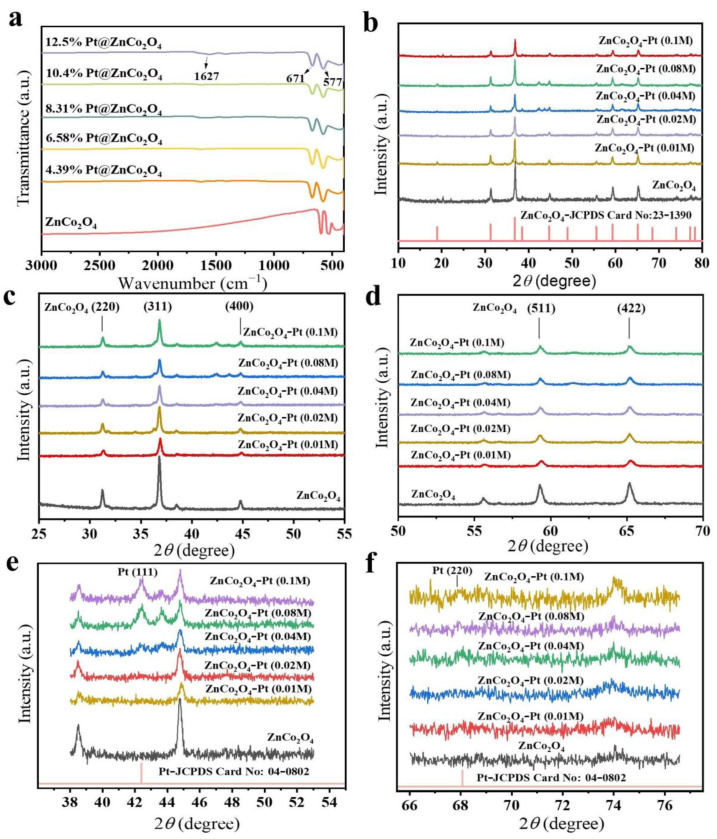
(**a**) FT-IR spectra and (**b**) XRD patterns of ZnCo_2_O_4_ microspheres with varying Pt loading levels. Comparison of XRD diffraction peaks on different crystal facets with (**c**,**d**) ZnCo_2_O_4_-JCPDS Card (No:23-1390) and (**e**,**f**) Pt-JCPDS Card (No: 04-0802).

Figure 3a presents FT-IR spectra of ZnCo_2_O_4_ microspheres and Pt@ZnCo_2_O_4_ microspheres with varying Pt loading concentrations. These spectra provide key insights into the impact of Pt loading on the structure and surface chemistry of the ZnCo_2_O_4_ material. First, all samples exhibit distinct absorption peaks at 671 cm^−1^ and 577 cm^−1^, which correspond to the characteristic vibrational modes of Zn-O and Co-O bonds, respectively, indicating that the primary matrix structure of the material is ZnCo_2_O_4_, and the spinel structure remains largely unchanged regardless of the Pt loading. This result suggests that Pt loading does not disrupt the crystal structure of ZnCo_2_O_4_, and the material retains its original oxide framework. Second, an absorption peak at 1627 cm^−1^ is observed in the spectra, with its intensity varied depending on the Pt loading concentration. The peak at 1627 cm^−1^ is typically attributed to the stretching vibrations of C=O or aromatic C=C bonds, but in oxide and metal-loaded materials, it may indicate surface defects or lattice distortions induced by Pt incorporation. Pt loading may alter the local electronic environment on the surface of ZnCo_2_O_4_, particularly charge distribution at the oxide surface and interface, leading to the appearance and intensity variation in this characteristic absorption peak at 1627 cm^−1^. This change can suggest the formation of new active sites on the material surface, potentially enhancing its catalytic performance. As the Pt loading increases from 0 to 12.5%, the fluctuation intensity of the 1627 cm^−1^ peak is gradually increased, which may be attributed to the dispersion of Pt nanoparticles on the surface of ZnCo_2_O_4_. Higher Pt loading may create more active centers on the ZnCo_2_O_4_ surface, increasing interface interactions that modulate the surface’s electronic properties. Furthermore, the introduction of Pt may facilitate electron migration on the ZnCo_2_O_4_ surface, optimizing the composite’s performance in catalytic applications. Therefore, the presence and intensity variation in the absorption peak further confirm that Pt loading induces changes in the surface chemical environment of ZnCo_2_O_4_. In summary, the infrared spectroscopy analysis of Pt@ZnCo_2_O_4_ materials with varying Pt loading demonstrates that Pt incorporation does not disrupt the underlying ZnCo_2_O_4_ structure, but it significantly affects the surface chemical environment. By controlling the Pt loading, the surface structure and electronic properties of the material can be well optimized, improving its performance in catalytic applications. These findings provide experimental evidence for the design and optimization of Pt@ZnCo_2_O_4_ composites in catalytic and other functional applications.

There is other evidence from X-ray diffraction (XRD) analysis on the composition and crystal structure of ZnCo_2_O_4_ and Pt@ZnCo_2_O_4_ microspheres with varying Pt loading concentrations. As shown in Figure 3b, the XRD patterns of ZnCo_2_O_4_ and Pt@ZnCo_2_O_4_ materials exhibit obvious diffraction peaks (2θ) at 18.965°, 31.254°, 36.827°, 38.527°, 44.786°, 55.624°, 59.323°, 65.197°, 68.588°, 74.076°, 77.294°, and 78.357°, which correspond to the (111), (220), (311), (222), (400), (422), (511), (440), (531), (620), (533), and (622) crystal planes, respectively, according to ZnCo_2_O_4_-JCPDS card (No. 23-1390). It can be clearly seen that the composition and crystal structure of ZnCo_2_O_4_ MSs remain essentially unchanged after Pt loading, which is shown in Figure 3c,d. In comparison with Pt-JCPDS Card (No: 04-0802), two characteristic peaks (2θ) at 42.307° and 67.868° for Pt are observed clearly, corresponding to (111) and (220) crystal planes, respectively, as shown in Figure 3e,f. Regardless of the Pt loading amount increased, no changes are observed in the XRD absorption peaks of the ZnCo_2_O_4_ substrate, further proving that Pt only exists on the surface of the microsphere without inducing any chemical bond interactions with ZnCo_2_O_4_. Combining with the elemental distribution data (Figure 2), it can be further concluded that Pt is successfully loaded onto the surface of the ZnCo_2_O_4_ microspheres in a highly dispersed or small particle size form without disrupting the spinel structure of the matrix. The crystal integrity of the ZnCo_2_O_4_ substrate is maintained during the Pt loading process, which makes it an effective support material for Pt. This structural stability ensures the long-term and stable performance of Pt@ZnCo_2_O_4_ materials in catalytic, sensing, and other applications.

**Figure 4:**
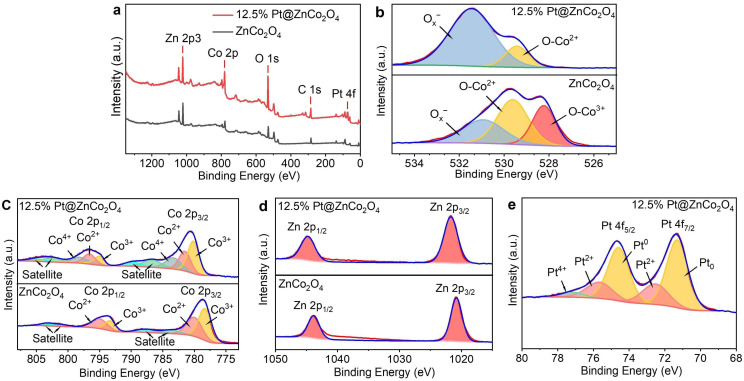
XPS spectra of ZnCo_2_O_4_ and 12.5% Pt@ZnCo_2_O_4_: (**a**) full spectrum, (**b**) O 1s, (**c**) Co 2p, (**d**) Zn 2p, (**e**) Pt 4f high-resolution spectra.

Moreover, the elemental valence state and surface property of ZnCo_2_O_4_ and 12.5% Pt@ZnCo_2_O_4_ microspheres were examined using X-ray photoelectron spectroscopy (XPS), as shown in Figure 4, providing an in-depth understanding of the changes on the surface chemical environment of ZnCo_2_O_4_ upon Pt loading. First, the XPS full spectrum (Figure 4a) clearly shows distinct Pt 4f orbital peaks at 71.37 eV and 74.68 eV in the Pt-doped sample, indicating the successful loading of Pt onto the surface of ZnCo_2_O_4_ microspheres. The O 1s high-resolution spectrum in Figure 4b further reveals a significant enrichment in surface oxygen species following Pt loading, particularly an increase in the content of adsorbed oxygen ions (such as O^−^ and O₂^−^). The increase in adsorbed oxygen also suggests the enrichment of oxygen vacancies on the material’s surface with high porosity. The presence of these oxygen vacancies generally enhances the adsorption capacity and catalytic activity toward substrate molecules, offering a potential advantage for Pt@ZnCo_2_O_4_ in catalytic applications. In the Co 2p high-resolution spectrum (Figure 4c), 12.5% Pt@ZnCo_2_O_4_ exhibits Co^2^⁺ 2p orbital peaks at 781.55 eV and 796.64 eV, while peaks at 780.14 eV and 794.93 eV correspond to Co^3^⁺ 2p orbitals. Additionally, Co⁴⁺ 2p orbital peaks are observed at 783.32 eV and 798.21 eV, indicating a change in the oxidation states of Co for ZnCo_2_O_4_ upon Pt loading. Further, satellite peaks at 786.74 eV, 789.74 eV, 805.07 eV, and 802.70 eV also support this observation. Compared to ZnCo_2_O_4_, the introduction of Pt leads to changes in the oxidation states of Co, which can be attributed to the electronic interactions between Pt and ZnCo_2_O_4_. In contrast, the Zn 2p high-resolution spectrum (Figure 4d) shows no significant changes in the Zn 2p orbital after Pt loading, suggesting that Pt loading primarily affects the electronic states of the Co element. In the Pt 4f high-resolution spectrum (Figure 4e), three distinct chemical states of Pt are observed, namely Pt⁰, Pt^2^⁺, and Pt⁴⁺. Notably, the majority of Pt exists in the monomorphic Pt^0^ state, implying that Pt is mainly distributed as monatomic Pt on the surface of ZnCo_2_O_4_ microspheres. The presence of monatomic Pt contributes to more catalytic active sites and greatly enlarges the catalytic activity of the material while preserving the original structure of ZnCo_2_O_4_.

In summary, the XPS analysis indicates that the Pt loading not only increases the surface concentration of adsorbed oxygen and oxygen vacancies but also significantly alters the variation in Co valence. Therefore, it might be their collective effect that contributes to the enhancement of the catalytic activity of Pt@ZnCo_2_O_4_ microspheres.

### 2.2. Investigation of Peroxidase-like Activity

#### 2.2.1. Comparison of Peroxidase-like Activities of Pt@ZnCo_2_O_4_ Microspheres with Different Pt Loading Levels

**Figure 5:**
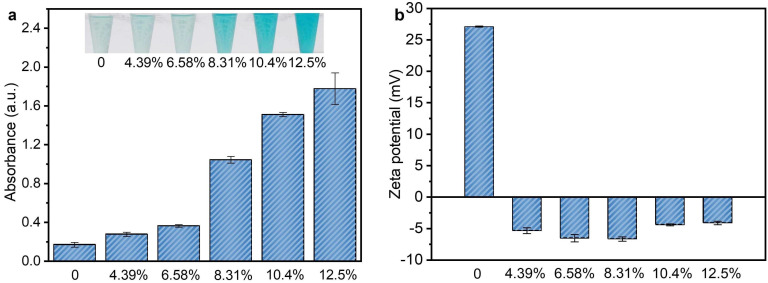
Enzyme-like activities (**a**) and Zeta potentials (**b**) of Pt@ZnCo_2_O_4_ microspheres with different Pt loading amounts (0~12.5%) in NaAc-HAc buffer solution (25 mM, pH 3.5) with addition of H_2_O_2_ (3.6 mM) and TMB (0.8 mM) at incubation temperature of 25 °C for 5 min; inset of (**a**) displays corresponding colorimetric images observed with the naked eyes.

The catalase-like activities of Pt@ZnCo_2_O_4_ MSs for six different Pt loading levels (0~12.5%) with the addition of H_2_O_2_ and TMB in NaAc-HAc buffer solution (pH 3.5) were examined. Figure 5a illustrates the peroxidase-like activities exhibited by different Pt@ZnCo_2_O_4_ MSs with varying Pt loading concentrations. It is worth noting that ZnCo_2_O_4_ microspheres demonstrate the lowest peroxidase-like activity under consistent experimental conditions. With the increase in Pt loading level, it is observed that the peroxidase-like activity of Pt@ZnCo_2_O_4_ gradually enhanced, particularly the 12.5% Pt@ZnCo_2_O_4_ MSs exhibits the highest activity, which is also evidenced by the above-mentioned FT-IR and XRD data in Figure 3. To assess the substrate adsorption capability of the mimetic enzymes, Zeta potential measurement of Pt@ZnCo_2_O_4_ MSs with varying Pt loading levels (0~12.5%) was also performed. As depicted in Figure 5b, the Zeta potentials of all Pt-doped ZnCo_2_O_4_ MSs are negatively charged, which are significantly lower than that of undoped ZnCo_2_O_4_ MSs. This reduction suggests that Pt loading induces changes in the charge distribution on the material’s surface, which may, in turn, affect its dispersibility and surface reactivity. The marked decrease in Zeta potential can also influence the material’s stability and adsorption characteristics, potentially improving its performance in catalytic reactions. In general, the materials with lower potentials are more likely to adsorb positively charged targets, leading to enhanced catalytic performance. For materials with similar zeta potentials, the amount of Pt loading becomes the primary factor affecting the enzyme-like activity.

As shown in Figure 5a,b, the peroxidase-like activity of Pt@ZnCo₂O₄ increases with the increased Pt contents, but after reaching 12.5%, it tends to approach equilibrium to a certain degree. By evaluating the atomic percentages of Zn, Co, and Pt in Pt@ZnCo₂O₄ synthesized with different concentrations of chloroplatinic acid solutions (Table S2), we find that as the Pt concentration increases, the atomic percentages of Zn and Co gradually decrease and stabilize while the Pt content reaches 10.4%, varying less until 12.5%, which is shown in Figure S5. Therefore, to obtain the enhanced peroxidase-like activity, the Pt content of 12.5% was selected as the most stable equilibrium state for the optimal atomic percentages of various component elements.

#### 2.2.2. Mechanism of Peroxidase-like Catalyzed by Pt@ZnCo_2_O_4_ Microspheres

**Figure 6:**
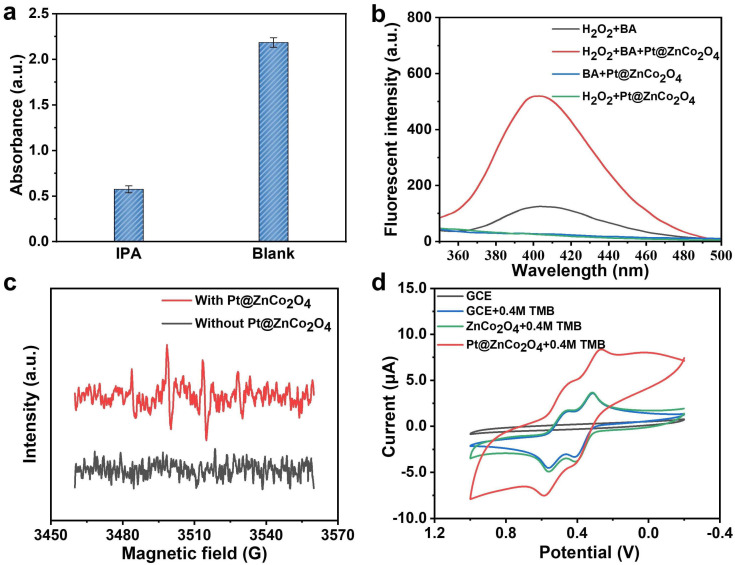
In 25 mM NaAc-HAc buffer solution (pH = 3.5): (**a**) absorbance at 652 nm with and without IPA of containing Pt@ZnCo_2_O_4_ (12.5%) + H_2_O_2_ (3.6 mM) + TMB (0.8 mM); (**b**) fluorescence spectra of different reaction systems, [BA] = 2.8 mM, [H_2_O_2_] = 4.0 mM, [Pt@ZnCo_2_O_4_ MSs] = 12.5%; (**c**) DMPO spin trapping EPR spectra with or without 12.5% Pt@ZnCo_2_O_4_, [DMPO] = 30 mM, [H_2_O_2_] = 2.8 mM; (**d**) cyclic voltammograms of different reaction systems with a saturated calomel electrode as the reference electrode, [Pt@ZnCo_2_O_4_ MSs] = 12.5%, [H_2_O_2_] = 4.0 mM, scan rate = 25 mV/s.

Under acidic conditions, peroxidase-like activity catalyzes the generation of •OH from H₂O₂, which can further oxidize the colorless TMB to produce blue oxTMB. To investigate the catalytic mechanism of Pt@ZnCo_2_O_4_ microspheres, isopropanol (IPA) was first chosen as a hydroxyl radical scavenger. As shown in Figure 6a, upon adding IPA, the absorbance at 652 nm decreased sharply compared to the blank group. Next, benzoic acid (BA) was used as a • OH-trapping probe. BA itself does not fluoresce, but upon oxidation by •OH, it generates hydroxybenzoic acid (OHBA), which exhibits fluorescence. The fluorescence spectra were measured with an excitation wavelength of 298 nm and an emission wavelength of 405 nm, as shown in Figure 6b. When only Pt@ZnCo_2_O_4_ microspheres and H_2_O_2_ are present, and there is no BA in the system (i.e., H_2_O_2_ + Pt@ZnCo_2_O_4_), or when only Pt@ZnCo_2_O_4_ microspheres and BA are present, and there is no H_2_O_2_ in the system (i.e., BA + Pt@ZnCo_2_O_4_), both no significant fluorescence signals can be measured. When Pt@ZnCo_2_O_4_ MSs are not used, and only H_2_O_2_ and BA are present in the system (i.e., H_2_O_2_ + BA), a weak fluorescence signal is produced. However, upon the addition of Pt@ZnCo_2_O_4_ MSs in the reaction system containing H_2_O_2_ and BA (i.e., H_2_O_2_ + BA + Pt@ZnCo_2_O_4_), a distinct characteristic peak is observed. These experimental results demonstrate that the Pt@ZnCo_2_O_4_ microspheres generate hydroxyl radical intermediates during the entire peroxidase-like catalytic process.

We further verified the generation of hydroxyl radicals through electron paramagnetic resonance (EPR), using 5,5-dimethyl-1-pyrroline N-oxide (DMPO) as a spin trapping agent, which can capture short-lived hydroxyl radicals and then form relatively stable DMPO/•OH spin adducts. As shown in Figure 6c, it can be clearly observed that when the system contains Pt@ZnCo_2_O_4_, a characteristic peak with a signal intensity of 1:2:2:1 appears in the EPR spectrum of the system. In the absence of Pt@ZnCo_2_O_4_, no characteristic peak of the DMPO/•OH spin adduct is observed. The above results demonstrate that Pt@ZnCo_2_O_4_ microspheres can catalyze the generation of •OH from H_2_O_2_ during the peroxidase-like catalytic process. Throughout the process, Pt@ZnCo_2_O_4_ first catalyzes the decomposition of H_2_O_2_ into •OH, which further catalyzes the oxidation of TMB to oxTMB. Therefore, the peroxidase reaction catalyzed by Pt@ZnCo_2_O_4_ microspheres conforms to the classical Fenton mechanism [32].

Additionally, electrochemical methods were employed to investigate the effect of electron transfer on the catalytic activity of Pt@ZnCo_2_O_4_ materials. The cyclic voltammetry (CV) technique was primarily used to examine the reaction processes of ZnCo_2_O_4_ and Pt@ZnCo_2_O_4_ microspheres with TMB in the absence of H_2_O_2_. This enabled the evaluation of the electrocatalytic oxidation behaviors of TMB on glassy carbon electrodes (GCEs) modified with ZnCo_2_O_4_ and Pt@ZnCo_2_O_4_ microspheres. Figure 6d shows the CV curves of the bare GCE, ZnCo_2_O_4_-modified GCE, and Pt@ZnCo_2_O_4_-modified GCE in a TMB (0.4 mM) containing 25 mM sodium acetate–acetic acid buffer solution (pH 3.5). The bare GCE shows no redox peaks in the buffer solution without TMB. In the presence of 0.4 M TMB, the bare GCE displays distinct reduction peaks at 0.56 V and 0.42 V, along with noticeable oxidation peaks at 0.32 V and 0.46 V. These oxidation and reduction peaks correspond to the characteristic redox behavior of TMB. Upon modification with ZnCo_2_O_4_ microspheres, the oxidation and reduction peak currents are slightly increased. When the GCE has been modified with Pt@ZnCo_2_O_4_ microspheres, both oxidation and reduction peak currents are significantly enhanced, with peak intensities much higher than those observed with the ZnCo_2_O_4_-modified electrode. Moreover, the active surface areas of ZnCo_2_O_4_^−^ and Pt@ZnCo_2_O_4_-modified GCEs are calculated as 1.310 × 10^−3^ cm^2^ and 2.973 × 10^−3^ cm^2^, respectively (as listed in Table S3 in the Supporting Information), indicating that Pt@ZnCo_2_O_4_ possesses larger surface area than that of ZnCo_2_O_4_. It suggests that Pt@ZnCo_2_O_4_ microspheres facilitate electron transfer more efficiently than ZnCo_2_O_4_ microspheres, leading to higher catalytic activity.

### 2.3. Enzyme Kinetics Study

**Figure 7:**
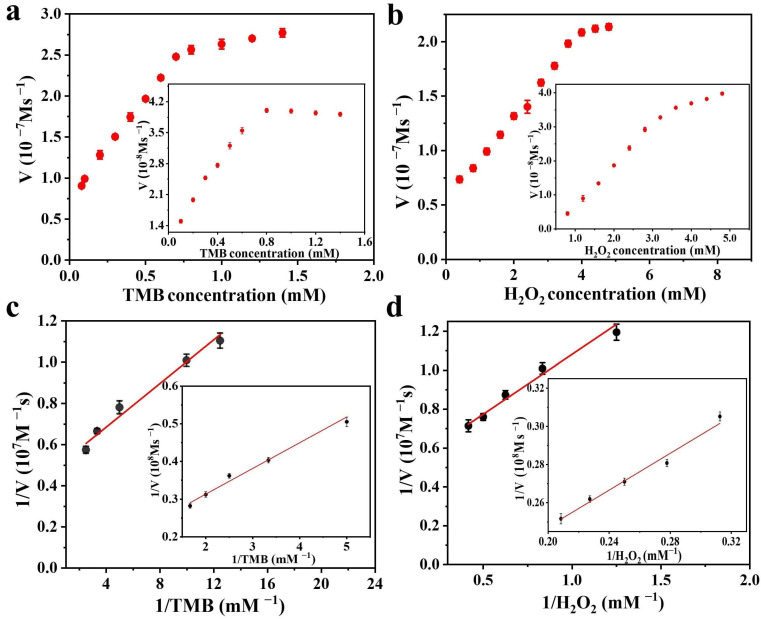
Steady-state kinetic analysis of 12.5% Pt@ZnCo_2_O_4_ microspheres: (**a**) Michaelis–Menten curves with TMB (while [H_2_O_2_] = 4.8 mM) and (**b**) H₂O₂ (while [TMB] = 1.0 mM) as the substrates; corresponding double reciprocal fitting plots for (**c**) TMB and (**d**) H₂O₂ as the substrates. Each inset is depicted for ZnCo_2_O_4_ microspheres.

The steady-state kinetics behavior of ZnCo_2_O_4_ and Pt@ZnCo_2_O_4_ microspheres as a peroxidase-like enzyme were investigated using TMB and H_2_O_2_ as substrates. The Michaelis–Menten curves and double reciprocal Lineweaver–Burk plots for ZnCo_2_O_4_ and 12.5% Pt@ZnCo_2_O_4_ microspheres with TMB and H₂O₂ as substrates are obtained, as shown in Figure 7. From these plots, the two main kinetic parameters, K_m_ and V_max_, were calculated, as listed in Table S1. The K_m_ values for Pt@ZnCo_2_O_4_ microspheres with TMB and H_2_O_2_ are 0.112 mM and 1.32 mM, respectively, and the Vmax values for Pt@ZnCo_2_O_4_ microspheres with TMB and H_2_O_2_ are 2.11 × 10^−7^ Ms^−1^ and 2.14 × 10^−7^ Ms^−1^, respectively, which are much superior to that of ZnCo_2_O_4_ microspheres. Moreover, the specific enzyme activity of Pt@ZnCo_2_O_4_ was compared with reported horseradish peroxidase (HRP) and different metal (like Pt, Co, Fe, etc.)-based peroxidase-like materials [33,34,35,36,37,38,39,40,41,42,43,44,45,46,47,48], as listed in Table 1. It can be seen that Pt@ZnCo_2_O_4_ microspheres exhibit much excellent substrate affinity and higher catalytic activity than other nanozymes.

### 2.4. Colorimetric Detection of L-Cys

#### 2.4.1. Principle of Detection

In this work, Pt@ZnCo_2_O_4_ microspheres function as peroxidase-like enzymes, catalyzing the decomposition of H_2_O_2_ into reactive oxygen species (ROS), specifically hydroxyl radicals (•OH). These •OH radicals subsequently oxidize the organic colorimetric substrate, TMB, leading to the formation of a coupling product. This coupled product exhibits an absorption peak at a specific wavelength in the visible region, resulting in a blue coloration. When L-Cys is introduced into the system, it inhibits the coupling reaction of the organic chromophore, thereby reflecting the level of L-Cys in the system through a decrease in the absorption value and a corresponding lightening of the color (Scheme 1).

#### 2.4.2. Investigation on Chromogenic Substrate and Colorimetric Feasibility

**Figure 8:**
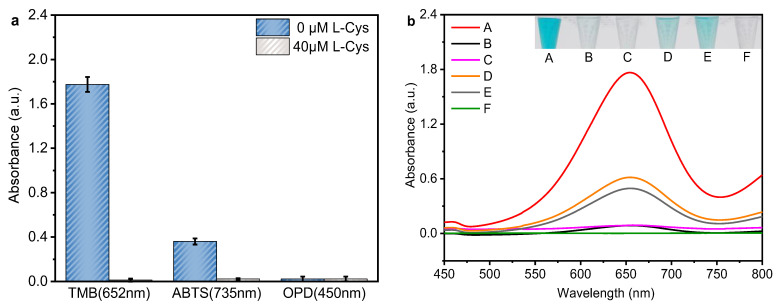
(**a**) Effect of chromogenic substrates on L-Cys detection while [H_2_O_2_] = 2.8 mM. (**b**) UV absorption spectra of different solution systems: (A) TMB + H_2_O_2_ + Pt@ZnCo_2_O_4_; (B) TMB + H_2_O_2_; (C) TMB + H_2_O_2_ + Pt@ZnCo_2_O_4_ + 40 μM L-Cys; (D) TMB + H_2_O_2_ + Pt@ZnCo_2_O_4_ + 4.0 μM L-Cys; (E) TMB + Pt@ZnCo_2_O_4_; (F) H_2_O_2_ + Pt@ZnCo_2_O_4_; insets of the colorimetric images observed with the naked eyes are included. The mixture was incubated at 40 °C for 10 min in 25 mM NaAc-HAc buffer solution (pH = 3.5), while [Pt@ZnCo_2_O_4_ MSs] = 12.5%, [TMB] = 0.8 mM, [H_2_O_2_] = 3.6 mM, and [L-Cys] = 40 μM.

With the best peroxidase-like activity, it is possible to employ the 12.5% Pt@ZnCo_2_O_4_ microspheres to establish a colorimetric sensor for visual detection of L-Cys. The effect of chromogenic substrates on the sensor’s signal output was investigated by comparing three common chromogenic substrates such as OPD, ABTS, and TMB. Under the same conditions, TMB exhibits the highest absorbance value as the chromogenic substrate (Figure 8a). When the target analyte, L-Cys, was added, L-Cys showed the most significant inhibitory effect on oxTMB, generating the strongest sensor signal. The possible reason for this is that, under weakly acidic conditions, TMB is more favorable for binding with Pt@ZnCo_2_O_4_ [49,50]. Therefore, TMB was selected as the optimal chromogenic substrate for further studies.

UV absorption tests were performed to verify the feasibility of the Pt@ZnCo_2_O_4_ microsphere-based colorimetric sensor for L-Cys detection. As shown in Figure 8b, curve A, with a very high absorption peak, represents the TMB and H_2_O_2_ system with Pt@ZnCo_2_O_4_ microspheres added, while curve B, with a much lower absorption peak, shows the TMB and H_2_O_2_ system without Pt@ZnCo_2_O_4_. It can be obviously observed that Pt@ZnCo_2_O_4_ microspheres exhibit high peroxidase-like activity. Curve C shows the addition of the target analyte, L-Cys, with a total concentration of 40 μM, to the system in curve A, causing a very large decrease in absorption peak. It is evident that when L-Cys is present at a certain concentration, the system turns colorless, and the characteristic absorption peak disappears. Thus, L-Cys inhibits the formation of oxTMB, resulting in a colorless system. When the added concentration of L-Cys is reduced to 4 μM (curve D), the system becomes light blue, and the characteristic absorption peak decreases significantly compared to Curve A but remains higher than that in the high-concentration L-Cys system (Curve C). This indicates that the inhibitory effect of L-Cys on oxTMB is related to its concentration. Curve E represents the system A without H_2_O_2_, i.e., TMB and Pt@ZnCo_2_O_4_. It can be seen that when only Pt@ZnCo_2_O_4_ material and TMB are present, system E shows a faint blue and a weak characteristic absorption peak, indicating that the material possesses some weak oxidase-like activity. However, its peroxidase-like activity is significantly larger than the oxidase-like activity. Then, the subsequent experiments were focused mainly on the peroxidase-like activity. Curve F shows the system with only Pt@ZnCo_2_O_4_ and H_2_O_2_. In the absence of the chromogenic substrate TMB, no signal is generated, confirming that the chromogenic substrate is a key factor influencing the performance of this colorimetric sensing system. Therefore, it is feasible to utilize Pt@ZnCo_2_O_4_ microsphere material for colorimetric detection of L-Cys.

#### 2.4.3. Optimization of Conditions

For a colorimetric detection system, the optimization of experimental parameters is of the utmost importance. In this work, several factors, including reaction time, temperature, pH, and concentrations of NaAc, TMB, and H_2_O_2_, were examined with two concentrations (4 µM and 40 µM) of L-Cys added (Figures S3 and S4). Figure S3a indicates that the absorbance difference (ΔA) of the detection system tends to stabilize at 20 min, and at the same time, the output signal of the colorimetric system is maximum and stable. Similar to the natural enzyme, the temperature has a great influence on enzyme activity, and the catalytic effect will be inhibited by both too-high and too-low reaction temperatures. As shown in Figure S3b, the Pt@ZnCo_2_O_4_ microspheres have the best catalytic effect and the largest sensing output signal at 50 °C. The simulated enzyme activity of Pt@ZnCo_2_O_4_ microspheres is closely related to the pH of the buffer solution. To obtain high detection sensitivity, the effect of buffer solutions with different pH on the sensing performance was investigated. Within the pH range of 3.0–5.0, relatively large sensing signals are observed, with a maximum at pH 3.5 (Figure S3c), indicating that the Pt@ZnCo_2_O_4_ microspheres have the optimal catalytic activity under acidic conditions. This is because peroxidase activity is determined through the mechanism of a Fenton-like reaction. One of the main factors influencing Fenton-like reagents is the pH value, which usually operates under acidic conditions and cannot catalyze the oxidation of H₂O₂ to produce hydroxyl radicals in neutral and alkaline environments [51,52]. Therefore, significant deviations from this pH value can affect the analysis of L-cysteine.

In addition, the detection performance was influenced by the concentration of the NaAc buffer solution, as shown in Figure S3d. It can be seen that the sensor system’s ΔA reaches its maximum at 652 nm when the NaAc concentration is 25 mM. It is worth noting that when the concentration of NaAc increases to 30 mM, its response in absorbance decreases so fast. Besides the inherent peroxidase-like activity, the buffer solution is an important external factor that can significantly alter the activity of nanozymes because buffering agents can interact with the particle surfaces [53]. Adding salts to buffer solutions can cause electrostatic interaction that affects enzyme kinetics [54]. Usually, excessively high salt concentrations in the buffer solution can affect the interaction between the nanozyme surface and the TMB and H₂O₂ substrates.

The concentration of the chromogenic substrate, TMB, plays an important role in the system’s detection performance. Figure S4a shows the sensor performance at different TMB concentrations. The best sensor signal is observed when the TMB concentration is 0.8 mM. Further increases in TMB concentration result in little to no change in ΔA, and even a slight decrease, because the catalytic sites on Pt@ZnCo_2_O_4_ microspheres are almost occupied by TMB molecules, and excessive substrate no longer reacts. The concentration of H_2_O_2_, acting as the electron acceptor in the catalytic reaction, also affects the catalytic oxidation rate of the chromogenic substrate. Figure S4b illustrates the sensor performance at different H_2_O_2_ concentrations. As the H_2_O_2_ concentration increases, ΔA gradually increases. When the H_2_O_2_ concentration reaches 3.6 mM, no further increase is observed, indicating that H_2_O_2_ has reached saturation and no longer participated in the reaction.

#### 2.4.4. Detection Performance

**Figure 9:**
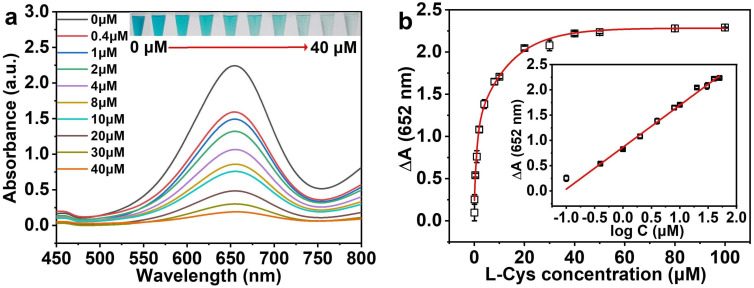
(**a**) UV-vis absorption spectra of the colorimetric system in 25 mM NaAc-HAc buffer solution (pH = 3.5) varied with the increased L-Cys concentration, including insets of the colorimetric images observed from blue to colorless with the naked eyes. (**b**) Response relation between ΔA_652nm_ and L-Cys concentration, with an inset of the corresponding calibration curve. [Pt@ZnCo_2_O_4_ MSs] = 12.5%, [TMB] = 0.8 mM, [H_2_O_2_] = 3.6 mM.

To evaluate the sensitivity of the colorimetric system for detecting L-Cys, the sensor’s responses to different concentrations of L-Cys were recorded under the optimal reaction conditions. As seen from an inset of Figure 9a, the solution color gradually changes from blue to colorless with the increased concentration of L-Cys in the system. When the L-Cys concentration exceeds 4 μM, the ΔA value becomes greater than 0.5, making it visibly distinguishable. When the L-Cys concentration exceeds 40 μM, the color and absorbance response signal remain relatively unchanged, indicating that the L-Cys concentration has reached saturation (Figure 9b). As shown in an inset of Figure 9b, the absorbance response value shows a good linear relationship with the logarithm of the L-Cys concentration (LogC_L-Cys_) in the range of 0.1~50 μM (R^2^ = 0.993). The limit of detection (LOD) for L-Cys was calculated to be 0.0163 μM using the 3σ/slope formula.

Additionally, the performance of the established Pt@ZnCo_2_O_4_ microspheres-based colorimetric sensor was compared with those previous methods for L-Cys detection based on metal-based mimetic enzyme nanomaterials reported elsewhere [55,56,57,58,59,60,61,62,63,64,65,66,67]. The summarized data are listed in Table 2. It can be seen that this work presents a relatively wider linear range and lower detection limit than others, indicating that the peroxidase-like colorimetric sensor possesses excellent sensing performance with high catalytic activity.

#### 2.4.5. Selectivity

**Figure 10:**
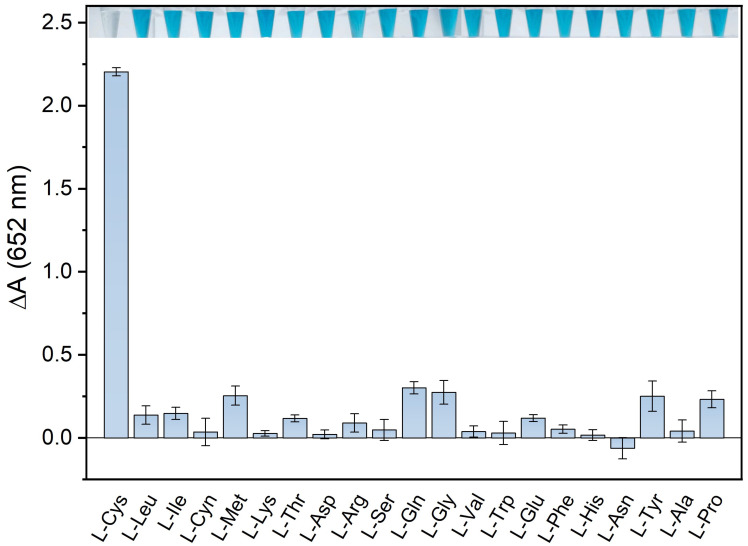
Selectivity of Pt@ZnCo_2_O_4_ microspheres for different amino acids in 25 mM NaAc-HAc buffer solution (pH = 3.5), including insets of the colorimetric images observed with the naked eye. [Pt@ZnCo_2_O_4_ MSs] = 12.5%, [TMB] = 0.8 mM, [H_2_O_2_] = 3.6 mM.

Selectivity is a crucial parameter for sensors. To evaluate the selectivity of the Pt@ZnCo_2_O_4_ microspheres in colorimetric detection of L-Cys, twenty other common amino acids were tested, including L-alanine (L-Ala), L-arginine (L-Arg), L-asparagine (L-Asn), L-aspartic acid (L-Asp), L-cysteine (L-Cys), L-glutamic acid (L-Glu), L-glutamine (L-Gln), L-glycine (L-Gly), L-histidine (L-His), L-isoleucine (L-Ile), L-leucine (L-Leu), L-lysine (L-Lys), L-methionine (L-Met), L-phenylalanine (L-Phe), L-proline (L-Pro), L-serine (L-Ser), L-threonine (L-Thr), L-tryptophan (L-Trp), L-tyrosine (L-Tyr), L-valine (L-Val), and L-cystine (L-Cyn). As shown in Figure 10, even when the concentrations of these interfering substances are ten times that of L-Cys, no significant effects from those amino acids on the detection of L-Cys can be observed. The colorimetric images of the twenty amino acids appear blue, while the L-Cys remains colorless in the reaction system, which is clearly observable with the naked eye, showing its good visual detection ability for L-Cys.

This is because, although the common twenty-one amino acids have similar structures and properties due to their amino and carboxyl groups, L-Cys differs significantly in structure from the other twenty amino acids due to the presence of a thiol group, which can form a relatively strong bonding interaction with the precious metal platinum. At the same time, in acidic environments such as pH 3.5, L-Cys exhibits a strong positive charge near its isoelectric point (pI = 5.07), indicating that its molecular ion carries a strong positive charge. According to the zeta potential data of Pt@ZnCo_2_O_4_ in Figure 5b, it can be inferred that its microsphere surface carries a negative charge, leading to strong electrostatic adsorption between L-Cys and the microsphere surface. Moreover, from the XPS data in Figure 4b, it can be seen that the loading of Pt increases the oxygen vacancies on the surface of Pt@ZnCo_2_O_4_ microspheres compared with ZnCo_2_O_4_ microspheres, as the oxygen vacancies increase the active sites on the surface of Pt@ZnCo_2_O_4_, thereby enhancing the electrostatic adsorption force between the negatively charged Pt@ZnCo_2_O_4_ surface and the positively charged cysteine molecule. Therefore, the Pt@ZnCo_2_O_4_ microspheres possess high selectivity in colorimetric detection of L-Cys against twenty other common amino acids.

In addition, the thiol compounds, such as methyl mercaptan, ethyl mercaptan, and phenylthiol, with no amino and carboxyl groups, did not affect the detection results. If the same concentration of cysteine is present in samples with different concentrations of other reactive oxygen species (peroxides), the analysis results will not be affected because the material in this work catalyzes the generation of hydroxyl radicals from hydrogen peroxide, oxidizing colorless TMB to blue oxTMB. The key step in detection is the reduction in oxTMB by L-cysteine, resulting in a difference in absorbance.

#### 2.4.6. Reusability and Usage Stability

**Figure 11:**
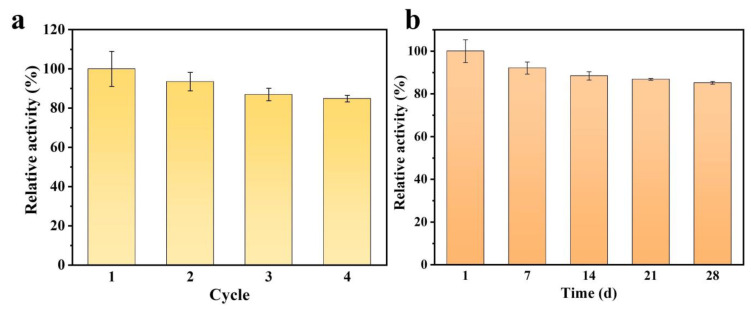
(**a**) Reusability and (**b**) storage stability of Pt@ZnCo_2_O_4_ microspheres in 25 mM NaAc-HAc buffer solution (pH = 3.5). [Pt@ZnCo_2_O_4_ MSs] = 12.5%, [TMB] = 0.8 mM, [H_2_O_2_] = 3.6 mM.

The usage stability of the material and its reusability are key factors affecting the performance of colorimetric sensors. As shown in Figure 11a, after four cycles of reuse, the peroxidase-like activity of the Pt@ZnCo_2_O_4_ microspheres still remains over 80%, demonstrating excellent reusability. Furthermore, the peroxidase-like activity of the Pt@ZnCo_2_O_4_ microspheres used for different periods was also evaluated. The results, shown in Figure 11b, indicate that after being dispersed in water for 28 d of usage, the activity of the material still remains above 85% when measured every seven days, suggesting good usage stability.

### 2.5. Analytical Application

The fabricated Pt@ZnCo_2_O_4_ microspheres-based colorimetric sensor was further applied to the detection of L-Cys in real samples like the L-Cysteine capsule, one of the health products of Pure Encapsulations. The result was compared with the content measured by HPLC (Table 3). As indicated in Table 3, the L-Cys contents measured by using the colorimetric method are in agreement with that of HPLC, that the recovery rates of L-Cys in real samples range from 96.9% to 103.7%, with relative standard deviation (RSD) ranging from 1.07% to 6.50%. Also, the recovery rates and RSD data both fall within a reasonable range, confirming the reliability of this colorimetric method for L-Cys in actual samples.

## 3. Materials and Methods

### 3.1. Reagents and Instruments

Glacial acetic acid (CH_3_COOH), hydrogen peroxide (H_2_O_2_, 30%), hydrochloric acid (HCl), anhydrous ethanol (CH_3_CH_2_OH), N,N-dimethylformamide (DMF), zinc acetate dihydrate (CH_3_COOZn·2H_2_O), cobalt acetate tetrahydrate (CH_3_COOCo·4H_2_O), benzoic acid (C_6_H_5_COOH), ethylene glycol (CH_2_OH)_2_, anhydrous sodium acetate (CH_3_COONa), and isopropanol (IPA) were purchased from Sinopharm Chemical Reagent Co., Ltd. (Shanghai, China). 3,3′,5,5′-Tetramethylbenzidine (TMB), polyvinylpyrrolidone (PVP), 5,5-dimethyl-1-pyrroline N-oxide (DMPO), and 2,2′-azinobis(3-ethylbenzothiazoline-6-sulfonic acid) diammonium salt (ABTS) were obtained from Aladdin Biochemical Technology Co., Ltd. (Shanghai, China). o-Phenylenediamine (OPD) was sourced from Yien Chemical Technology Co., Ltd. (Shanghai, China). Potassium chloroplatinate (K_2_PtCl_4_) was acquired from McLean Biochemical Technology Co., Ltd. (Shanghai, China). Nitric acid (HNO_3_) was procured from Kelong Chemical Reagent Factory (Sichuan, China). Aluminum oxide powder (Al_2_O_3_, 1.0, 0.1, and 0.05 um) was obtained from Xianren Instrument Co., Ltd. (Shanghai, China). L-Cysteine capsule, one of Pure Encapsulations, was sourced from Puritan’s Pride Health Food Co., Ltd. (New York, NY, USA). Distilled water (conductivity ≥ 18.2 MΩ·cm) from Watsons Food and Beverage Co., Ltd. (Guangzhou, China) was used for sample preparation and testing in the experiments.

Scanning electron microscopy (SEM, MIRA4) was utilized for morphological and compositional analysis of the samples, with equipment sourced from Tescan (Brno, Czech Republic). The Zeta potential was measured using a NanoBrook 90Puls Zeta potential and laser particle size analyzer. Powder X-ray diffraction (PXRD) was performed using a Smart Lab SE powder diffractometer from Rigaku Corporation (Akishima-shi, Japan). X-ray photoelectron spectroscopy (XPS) analysis was conducted on a Thermo Scientific K-Alpha instrument from Thermo Fisher Scientific (Waltham, MA, USA). Fourier transform infrared spectroscopy (FT-IR) was performed using a Thermo Scientific Nicolet iS20 spectrometer (Thermo Fisher Scientific, Waltham, MA, USA). Free radicals were assessed using a Bruker EMXplus-6/1 electron paramagnetic resonance (EPR) spectrometer from Bruker Corporation (Bremen, Germany). High-temperature carbonization of the samples was carried out in an HLG-X-12 graphite furnace from Hengli (Luoyang, China). Ultraviolet (UV) spectroscopy measurements were conducted using a UV-1900i UV spectrophotometer from Shimadzu Corporation (Kyoto, Japan). Fluorescence spectroscopy was performed on an F-7000 fluorescence spectrophotometer from Hitachi (Tokyo, Japan). Electrochemical measurements were carried out using a CHI 660E electrochemical workstation from Shanghai Chenhua Instrument (Shanghai, China).

### 3.2. Synthesis of ZnCo_2_O_4_ Microspheres

The Zn-Co carbonate precursor was synthesized using a co-precipitation method. A total of 0.6585 g of Zn(CH_3_COO)_2_·2H_2_O and 1.4946 g of Co(CH_3_COO)_2_·4H_2_O were weighed and placed in a 250.0 mL round-bottom flask, followed by the addition of 150.0 mL of ethylene glycol. The resulting mixture was vigorously stirred for 1 h, after which the temperature was gradually increased to 50 °C while maintaining stirring for 30 min. Subsequently, the mixture was transferred to an oil bath and heated to 170 °C at a stirring speed of 100 rpm, allowing for condensation reflux for 2 h. Upon cooling to room temperature, the precipitate was collected by centrifugation. The resulting purple precipitate was washed six times with ethanol and then centrifuged to collect the product. The collected precipitate was dried at 60 °C for 12 h. The synthesized Zn-Co carbonate precursor was then placed in an aluminum chloride crucible and subjected to annealing at 600 °C for 5 h, with a heating rate of 5 °C/min in a tube furnace. After annealing, the solid is directly obtained from the aluminum chloride ceramic crucible and thoroughly ground to obtain the ZnCo_2_O_4_ microspheres.

### 3.3. Synthesis of Pt@ZnCo_2_O_4_ Microspheres

Fifty milligrams of ZnCo_2_O_4_ MSs and thirty milligrams of polyvinylpyrrolidone (PVP) were dissolved in 50 mL of ethylene glycol. Subsequently, 0.6 mL of a 0.02 M K_2_PtCl_4_ aqueous solution was added to the mixture. The solution was then heated to 110 °C and maintained at this temperature for 2 h. After the reaction, the precipitate was collected by centrifugation and washed several times with deionized water and ethanol. The product was dried in an oven at 60 °C for 12 h. Additional K_2_PtCl_4_ aqueous solutions were prepared with concentrations of 0.01 M, 0.04 M, 0.08 M, and 0.10 M to synthesize different Pt@ZnCo_2_O_4_ MSs with varying amounts of Pt doped (Table S2). Usually, the microspheres were stored dry at 4 °C and dispersed in water during use.

### 3.4. Measurement of Catalase-like Activity

Five microliters of suspensions (5 mg/mL) of the above-synthesized Pt@ZnCo_2_O_4_ MSs were added to 455 μL of a 25 mM sodium acetate–acetic acid buffer solution (pH 3.5). Subsequently, 20 μL of a 70 mM hydrogen peroxide solution and 20 μL of a 20 mM 3,3′,5,5′-tetramethylbenzidine (TMB) solution were added. The mixture was incubated in a constant temperature metal bath at 25 °C for 5 min. The absorbance values at 652 nm and 480 nm were measured using UV-vis spectroscopy, and the difference in absorbance (A_652_–A_480_) was obtained.

### 3.5. Measurement of Catalytic Mechanism

(1) Fluorescence measurement of hydroxyl radicals (·OH): Benzoic acid (BA), which has no fluorescence properties, was selected as the probe to trace the hydroxyl radicals (·OH) generated by the Pt@ZnCo_2_O_4_ microspheres. In a 1.5 mL centrifuge tube, 10 μL of 1.0 mg/mL 12.5% Pt@ZnCo_2_O_4_ MSs, 100 μL of 14 mM BA, and 20 μL of 100 mM hydrogen peroxide were added. The solution was then diluted to a total volume of 500 μL with a 25 mM sodium acetate–acetic acid buffer (pH 3.5). The mixture was homogenized using a vortex mixer and incubated in a constant temperature metal bath at 37 °C for 30 min. The fluorescence intensity of the system was measured, with the excitation wavelength (Ex) set at 298 nm and the emission wavelength (Em) at 405 nm.

(2) Isopropanol (IPA) for hydroxyl radical (·OH) capture: For the IPA group, 5 μL of 1.0 mg/mL 12.5% Pt@ZnCo_2_O_4_ MSs, 20 μL of 90 mM hydrogen peroxide (H_2_O_2_), 100 μL of isopropanol (IPA), and 20 μL of 20 mM TMB were added to a 1.5 mL centrifuge tube. The total volume of the solution was then adjusted to 500 μL with a 25 mM sodium acetate–acetic acid buffer (pH 3.5). For the control group without IPA, the same procedure was followed, without adding IPA.

(3) Electrochemical testing: 1.0 mg of 12.5% Pt@ZnCo_2_O_4_ MSs was dissolved in 1 mL of distilled water and sonicated for 30 min. Afterward, 5 μL of this dispersion was dropped onto a glassy carbon electrode. The electrode was allowed to air dry before being immersed in a mixed solution containing 25 mM sodium acetate–acetic acid buffer (pH 3.5) and 0.4 mM TMB, maintaining a total volume of 5 mL for the reaction system. The electrochemical test was conducted using cyclic voltammetry (CV) with a three-electrode setup, scanning from −0.2 V to 1.0 V at a scan rate of 0.025 V/s.

(4) Electron paramagnetic resonance experiment: A reaction was carried out at room temperature in the dark using a 25 mM sodium acetate–acetic acid buffer (pH 3.5) containing 10 μg/mL of 12.5% Pt@ZnCo_2_O_4_ MSs, 2.8 mM H_2_O_2_, and 30 mM DMPO. After a reaction time of 5 min, the production of radicals in the system was detected using an EPR spectrometer. According to the Randles–Sevcik equation [68], with a scan rate of 25 mV/s (n = 1 and D = 7×10^−6^ cm^2^/s), the effective specific surface area of the series of ZnCo₂O₄ and Pt@ZnCo₂O₄ modified electrodes were calculated.

I_p_ = 2.69 × 10^5^n^3/2^AD^1/2^Cv^1/2^ (1)

Here, I_p_ is the peak current, n is the number of electrons transferred, A is the effective specific surface area of the electrode, and D is the diffusion coefficient.

### 3.6. Kinetics Measurement of Peroxidase-like Enzymes

Enzyme kinetics experiment: The experiment was conducted in a 25 mM sodium acetate–acetic acid buffer (pH 3.5) containing 10 μg/mL of 12.5% Pt@ZnCo_2_O_4_ MSs. When the concentration of H_2_O_2_ in the fixed system was set at 4.8 mM, the concentration of TMB varied between 0.1 mM and 1.4 mM. Conversely, when the concentration of TMB was fixed at 1.0 mM, the concentration of H_2_O_2_ varied from 0.4 mM to 4.4 mM. The absorbance at the maximum wavelength (652 nm) was measured every 10 s for the first 150 s of the reaction. Subsequently, a plot of 1/[V] versus 1/[S] was constructed to calculate the Michaelis constant (K_m_) and the maximum reaction velocity (V_max_) for the 12.5% Pt@ZnCo_2_O_4_ MSs. All experiments were repeated three times. According to Lambert–Beer’s law, the substrate concentration can be calculated using the following formula:

A = εbc(2)

where A represents the absorbance; ε denotes the molar absorptivity coefficient, with the molar absorptivity of TMB in this system being 39,000 M^−1^cm^−1^; b signifies the path length, measured in centimeters, with a consistent value of 1 cm in this experiment; and c refers to the substrate concentration, expressed in mol/L.

The relevant kinetic parameters were calculated using the Lineweaver–Burk plot along with the Michaelis–Menten equation, as follows:

(3) $$\frac{1}{V}=\frac{1}{\left[S\right]}\cdot \frac{K_{m}}{V_{max}}+\frac{1}{V_{max}}$$

In this context, V represents the initial velocity, V_max_ denotes the maximum reaction velocity, [S] indicates the substrate concentration, and K_m_ corresponds to the Michaelis constant.

### 3.7. Procedure for Colorimetric Detection of L-Cysteine

(1) Selection of different chromogenic substrates: A total of 5 μL of 1.0 mg/mL 12.5% Pt@ZnCo_2_O_4_ MSs, 20 μL of 70 mM H_2_O_2_, and 20 μL of 20 mM TMB were mixed into 455 μL of 25 mM NaAc–HAc buffer (pH 3.5) and reacted at 40 °C for 10 min. In the experimental group, 20 μL of 1 mM L-Cys was added while keeping the other components unchanged, maintaining a final volume of 500 μL. The chromogenic substrates were then varied to OPD and ABTS. The absorbance differences between the blank and experimental groups for different chromogenic substrates were measured and calculated as ΔA′ = ΔA_Blank_ (A_652_ − A_480_) − ΔA_L-Cys_ (A_652_ − A_480_).

(2) Feasibility analysis: A total of 5 μL of 1.0 mg/mL 12.5% Pt@ZnCo_2_O_4_ MSs, 20 μL of 90 mM H_2_O_2_, and 20 μL of 20 mM TMB were mixed into 455 μL of 25 mM NaAc-HAc buffer (pH 3.5) and incubated at 40 °C for 20 min. The absorption spectrum was then measured in the range of 450 nm to 800 nm. In the sample group, 5 μL of 1.0 mg/mL 12.5% Pt@ZnCo_2_O_4_ MSs, 20 μL of 1.0 mM L-Cys, 20 μL of 90 mM H_2_O_2_, and 20 μL of 20 mM TMB were mixed into 435 μL of 25 mM NaAC–HAc buffer (pH 3.5).

(3) Optimization of testing conditions: Multiple parameters affecting the colorimetric detection of L-Cys were investigated, including reaction time, buffer pH, buffer concentration, reaction temperature, TMB concentration, and H_2_O_2_ concentration. Each experimental system had a total volume of 500 μL, with 5 μL of 1 mg/mL 12.5% Pt@ZnCo_2_O_4_ MSs and 20 μL of 1.0 mM L-Cys. The volumes of TMB and H_2_O_2_ added were both 20 μL, with their final concentrations indicated accordingly. The components were added in the following order: buffer, 12.5% Pt@ZnCo_2_O_4_, L-Cys, H_2_O_2_, and TMB.

(4) Colorimetric detection of L-Cys: The total volume of the system was maintained at 500 μL. Under optimal conditions, the blank group was set without the target substance L-Cys, while the experimental groups contained a series of different concentrations of L-Cys. The absorbances at 652 nm and 480 nm were measured for both blank group and each experimental group, with the calculation of ΔA as follows: ΔA = ΔA_Blank_ (A_652_ − A_480_) − ΔA_L-Cys_ (A_652_ − A_480_).

### 3.8. Selectivity Measurement

Selecting other amino acids as interferents: Other amino acids were added as interferents to the colorimetric detection system. The absorbances at 652 nm and 480 nm were measured, and the differences in absorbance were compared under the presence of different amino acids using the equation: ΔA = ΔA_Blank_ (A_652_ − A_480_) − ΔA_amino acid_ (A_652_ − A_480_).

### 3.9. Procedure for Detection of L-Cysteine in Actual Samples

Sample pretreatment: Dissolving 10 mg of L-Cysteine capsule powder in 10 mL of NaAC–HAc buffer (pH = 3.0) and then filtering the solution through a membrane to remove insoluble substances after centrifugation, the filtrate was collected and diluted 100 times.

Actual sample detection: Taking 5 μL of 1 mg/mL 12.5% Pt@ZnCo_2_O_4_, 20 μL of 90 mM H_2_O_2_, 20 μL of 20 mM TMB, and 20 μL of L-Cysteine sample solution added into 435 μL of 25 mM NaAc–HAc buffer (pH = 3.5) and mixed thoroughly, after reacting at 50 °C for 20 min, the absorbances at 652 nm and 480 nm were measured.

Examination of reusability and storage stability of Pt@ZnCo_2_O_4_ microspheres: First, 5 μL of 1 mg/mL 12.5% Pt@ZnCo_2_O_4_, 20 μL of 90 mM H_2_O_2_, and 20 μL of 20 mM TMB were added into 455 μL of 25 mM NaAC–HAc buffer (pH = 3.5) and mixed thoroughly. After reacting at 50 °C for 10 min, the mixture was centrifuged at 12,000 rpm for 5 min. Then, the absorbances of the supernatant were measured to obtain ΔA (A_652_ − A_480_). Finally, the remaining material was washed with distilled water three times and reused; then, 1 mL of 1.0 mg/mL 12.5% Pt@ZnCo_2_O_4_ was prepared, its absorbance ΔA (A_652_ − A_480_) was measured in the above catalytic system every seven days, and the relative activity was calculated referring to the highest absorbance as 100% activity for comparison.

## 4. Conclusions

In this work, a one-pot method was employed to synthesize Pt@ZnCo_2_O_4_ microspheres with the same morphology but varying Pt-loading levels. The peroxidase-like activities of these materials were systematically investigated. Compared to the spinel-structured ZnCo_2_O_4_ microspheres, the Pt@ZnCo_2_O_4_ microspheres exhibited slightly larger particle sizes, while their structural integrity remained largely unchanged. The incorporation of Pt^0^ increased the oxygen vacancies in the ZnCo_2_O_4_ microspheres, enhancing their substrate affinity and catalytic activity. The interaction between Pt@ZnCo_2_O_4_ microspheres and TMB through electrostatic adsorption accelerated the electron transfer throughout the catalytic process, with the peroxidase-like catalysis operating via a Fenton mechanism. Kinetic studies demonstrated that the Km values for both substrates (TMB and H_2_O_2_) with Pt@ZnCo_2_O_4_ microspheres were lower than the reported Km value of horseradish peroxidase, indicating superior catalytic activity. The L-cysteine colorimetric sensor based on Pt@ZnCo_2_O_4_ microspheres displayed high sensitivity, with a detection limit of 0.0163 μM. Furthermore, this material demonstrated good reusability and usage stability. The established sensing method offers the advantages of good accuracy and simplicity, successfully detecting L-cysteine in health supplements with a recovery rate of 96.9% to 103.7%. This work not only expands the horizons for the application of ZnCo_2_O_4_ spinel materials in bioanalytical chemistry but also offers valuable insights into the design and development of novel noble metal-loading artificial mimetic enzymes, showing significant practical potential.

## Figures and Tables

**Table 1:** Comparison of K_m_ and V_max_ with HRP and different metal-based nanozyme materials.

Catalyst	Km (mM)		Vmax (10^−8^ Ms^−1^)		References
	TMB	H_2_O_2_	TMB	H_2_O_2_	
HRP	0.434	3.70	10.0	8.71	[33]
Fe_3_O_4_/LNPs	0.510	5.30	1.03	0.96	[34]
Fe_3_O_4_ QDs	0.030	31.1	137	8.89	[35]
MgFe_2_O_4_MNPs	0.670	4.61	2.09	13.5	[36]
Co_3_O_4_ NDs	0.430	5.29	350	839	[37]
Co_3_O_4_-CeO_2_ NSs	0.420	138	13.7	3.98	[38]
CePO_4_-CeO_2_	0.230	4.76	8.78	29.8	[39]
CoTCPP-TiO_2_	0.011	1.49	9.56	4.73	[40]
CeO_2_/Co_3_O_4_ HNs	0.495	1.23	27.1	8.87	[41]
Cu-BDC@FeMo_6_	1.81	0.69	10.5	7.12	[42]
Pd@PtBi_5_	1.67	7.24	10.5	4.39	[43]
BiO-BDC-NH_2_	0.410	7.19	5.81	3.85	[44]
MOF-818	20.4	58.6	7.85	6.66	[45]
CuO/Pt NSs	0.413	2.89	14.6	8.85	[46]
Ag/Pt NCs	1.08	42.8	2.08	1.36	[47]
Pt/EMT zeolite	0.160	0.58	4.72	11.6	[48]
12.5% Pt@ZnCo_2_O_4_	0.112	1.32	21.1	21.4	This work

Note: BDC—Benzene dicarboxylic acid; CoTCPP—cobalt (II) 5,10,15,20-Tetrakis (4-carboxylpheyl)porphyrin; EMT—a kind of low-silica microporous aluminosilicate material; HNs—hollow nanocubes; HRP—horseradish peroxidase; LNP—magnetic nanoparticles; NCs—nanoclusters; NDs—nanodisks; NSs—nanosheets; MNPs—metal nanoparticles; MOF—metal–organic framework; QDs—quantum dots.

**Table 2:** Comparison of L-Cys detection with different metal-based mimetic enzymes.

Metal-Based Mimetic Enzymes	Analytical Method	Linear Range (μM)	Limit of Detection (μM)	References
Fe_3_O_4_ nanofibers	Colorimetric	2–10	0.0280	[55]
Van-Pt_1_/Pd_1_ NPs	Colorimetric	6–100	0.07	[56]
PtCo@MWCNTs	Colorimetric	0.01–60.0	0.041	[57]
PBNCs	Colorimetric	5–9	2.00	[58]
TAnc-Mn*_x_*_-*y*_	Colorimetric	8.26–90.86	2.28	[59]
CoO/Co-Try-GQD	Colorimetric	0.05–2	0.032	[60]
CeCuO_x_/C	Colorimetric	50–900	3.24	[61]
Co-N/C	Colorimetric	1–30	0.55	[62]
AgNPs	Colorimetric	0.5–20	0.0904	[63]
U66-PV-Pep@AuNP	Colorimetric	2.5–40.0	0.33	[64]
Pt/MnO_2_/GO	Colorimetric	0.5–32	0.12	[65]
GAP	Colorimetric	0.01–20.0	0.094	[66]
MNB/PC	Colorimetric	0–35	0.10	[67]
12.5% Pt@ZnCo_2_O_4_	Colorimetric	0.1–50	0.0163	This work

Note: GAP—Guanosine-5′-triphosphate; GQD—graphene quantum dots; GO—Graphene oxide; MNB/PC—MnO_2_ nanobelts anchoring on porous carbon; MWCNTs—multi-walled carbon nanotubes; PBNCs—Prussian blue nanocubes; TAnc—tannic acid enzyme mimics; U66-PV-Pep—UiO-66-NH_2_-poly (dimethylvinyloxazolinone)-pepsin; Van—vancomycin.

**Table 3:** Determination of L-Cys content in health product capsule (*n* = 3).

Sample	HPLC (μM)	This Method (μM)	Spiked (μM)	Measured (μM)	Recovery (%)	RSD (%)	RD (%)
L-Cysteine Capsule	7.962	8.055	5.000	13.24	103.7	1.07	1.2
			10.00	17.74	96.9	6.50	
			15.00	22.82	98.4	1.30	

## Data Availability

Data will be made available upon request.

## Supplementary Materials

The following supporting information can be downloaded at: https://www.mdpi.com/article/10.3390/molecules30010187/s1, Figure S1. Particle size distribution of ZnCo_2_O_4_ microspheres formed by aggregation. Figure S2. N₂ adsorption–desorption isotherm and pore size distribution diagram of ZnCo_2_O_4_. Figure S3. Effect of reaction time (a), temperature (b), pH (c), and NaAc concentration (d) on the colorimetric sensor for detecting L-Cys. Figure S4. Effect of TMB (a) and H_2_O_2_ concentrations on the detection of L-Cys using the colorimetric sensor. Figure S5. Atomic percentages of Co, Zn, and Pt in 4.39% Pt@ZnCo_2_O_4_, 6.58% Pt@ZnCo_2_O_4_, 8.31% Pt@ZnCo_2_O_4_, 10.4% Pt@ZnCo_2_O_4_, and 12.5% Pt@ZnCo_2_O_4_ with corresponding concentrations of chloroplatinic acid solutions added (0.01 M, 0.02 M, 0.04 M, 0.08 M, and 0.10 M). Table S1. Comparison of K_m_ and V_max_ values between ZnCo_2_O_4_ microspheres and 12.5% Pt@ZnCo_2_O_4_ microspheres. Table S2. Contents of metal elements in Pt@ZnCo_2_O_4_ microspheres at varying levels of Pt loaded. Table S3. Active surface area of different modified electrodes.

## References

[1] Ye J., Du J., Wang A., Yang Y., Zhu J., Li W., Wan C., Yin F., He G., Chen H. (2024). Reduced spinel oxide ZnCo_2_O_4_ with tetrahedral Co^2+^ sites for electrochemical nitrate reduction to ammonia and energy conversion. Chem. Eng. J..

[2] Zhang X., Zhang Y., Tian J., Guo Y., Zhou Z., Liu Z., Zhao Z., Liu B., Li J. (2024). Generating ^1^O_2_ and Co^IV^=O through efficient peroxymonosulfate activation by ZnCo_2_O_4_ nanosheets for pollutant control. Nano Res..

[3] Xiao K., Wang Y., Wu P., Hou L., Liu Z.-Q. (2023). Activating lattice oxygen in spinel ZnCo_2_O_4_ through filling oxygen vacancies with fluorine for electrocatalytic oxygen evolution. Angew. Chem. Int. Ed..

[4] Deng W., Wang X., Hao X., Jin Z. (2024). Embedding ZnCo_2_O_4_ quantum dots onto graphdiyne (g-C*_n_*H_2*n-*2_) nanosheets as a 0D/2D S-scheme heterojunction for highly efficient photocatalytic H_2_ evolution. Sep. Purif. Technol..

[5] Ganesan M., Alagar S., Bagchi V., Piraman S. (2024). Surface oxygen engineered ZnCo_2_O_4_ planar hybrid supercapacitor electrode for high energy applications. J. Energy Storage.

[6] Geng W., Song P., Duan L., Luan T. (2025). MOFs-derived In_2_O_3_ hollow microtubes/ZnCo_2_O_4_ microflowers for fast and sensitive detection of *n*-butanol. Sens. Actuators B Chem..

[7] Huang J.-Y., Li H.-J., Li L.-X., Chen R., Liu F., Wu L., Feng Z.-M., Yin Y.-L., Cao Z., Yu D. (2024). Sensitive detection of H_2_S based on Ce doped ZnCo_2_O_4_ hollow microspheres at low working temperature. Anal. Methods.

[8] Wu J., Zhang X., Yu F., Tian M., Wang Y., Wu J., Lu W., Guo Y. (2024). Exploration of cobalt-based spinel oxide nanocatalysts MCo_2_O_4_ (M = Mn, Fe, Co, Ni, Cu, Zn) for glucose electrochemical sensing: NiCo_2_O_4_ exhibits largest Faradaic current. Chem. Eng. J..

[9] Chen J., Wei X., Tang H., Munyemana J.C., Guan M., Zhang S., Qiu H. (2021). Deep eutectic solvents-assisted synthesis of ZnCo_2_O_4_ nanosheets as peroxidase-like nanozyme and its application in colorimetric logic gate. Talanta.

[10] Chu Y., Yin D., Wang Z., Li G., Wang Y., Liu Q., Yue K. (2025). Porphyrin modified ZnCo_2_O_4_ nanospheres as the excellent peroxidase/oxidase dual nanozymes for colorimetric sensing of cholesterol. Colloids Surf. A Physicochem. Eng. Asp..

[11] Yin D., Yang H., Wang S., Yang Z., Liu Q., Zhang X., Zhang X. (2020). Ce-doped ZnCo_2_O_4_ nanospheres: Synthesis, double enzyme-like performances, catalytic mechanism and fast colorimetric determination for glutathione. Colloids Surf. A Physicochem. Eng. Asp..

[12] Yu K., Lou L.-L., Liu S., Zhou W. (2020). Asymmetric oxygen vacancies: The intrinsic redox active sites in metal oxide catalysts. Adv. Sci..

[13] Cao Z., Tang Y., Zou H.-Y., Huang Y., Wu L., Xiao Z.-L., Feng Z.-M., Yin Y.-L., Yu D. (2022). Long-term and stable detection of H_2_S in a pig house at low operating temperature based on Ce_2_O_3_/In_2_O_3_ hollow microspheres with a remote monitoring system. Sens. Actuators B Chem..

[14] Li H., Hansen L., Aliyeva A., Wang J., Qiu H., Müller M., Chen S., Aktas C., Kienle L., Hartke B. (2025). Plasma-engineering of Pt-decorated NiCo_2_O_4_ nanowires with rich oxygen vacancies for enhanced oxygen electrocatalysis and zinc-air battery performance. Appl. Catal. B Environ. Energy.

[15] Ma Y., Chen M., Huang K., Chang W. (2024). The impact of cysteine on lifespan in three model organisms: A systematic review and meta-analysis. Aging Cell.

[16] Cramer S.L., Saha A., Liu J., Tadi S., Tiziani S., Yan W., Triplett K., Lamb C., Alters S.E., Rowlinson S. (2017). Systemic depletion of L-cyst(e)ine with cyst(e)inase increases reactive oxygen species and suppresses tumor growth. Nat. Med..

[17] Himmelfarb J., McMenamin E., McMonagle E. (2002). Plasma aminothiol oxidation in chronic hemodialysis patients. Kidney Int..

[18] Sigit J.I., Hages M., Brensing K.A., Frotscher U., Pietrzik K., Bergmann K.V., Lütjohann D. (2001). Total plasma homocysteine and related amino acids in end-stage renal disease (ESRD) patients measured by gas chromatography-mass spectrometry–comparison with the Abbott IMx homocysteine assay and the HPLC method. Clin. Chem. Lab. Med..

[19] Pervez W., Laraib;Yin C., Huo F. (2025). Homocysteine fluorescent probes: Sensing mechanisms and biological applications. Coord. Chem. Rev..

[20] Zou W., Wang Q., Gong F., Kuang Y., Xia J., Cao Z. (2019). Polymer nanoparticles integrated with ESIPT modules for sensing cysteine based on modulation of their tautomeric emission. Anal. Methods.

[21] Chen X., Gong F., Cao Z., Zou W., Gu T. (2018). Highly cysteine-selective fluorescent nanoprobes based on ultrabright and directly synthesized carbon quantum dots. Anal. Bioanal. Chem..

[22] Chand R., Kumar Jha S., Islam K., Han D., Shin I.-S., Kim Y.-S. (2013). Analytical detection of biological thiols in a microchip capillary channel. Biosens. Bioelectron..

[23] Li Z.P., Duan X.R., Liu C.H., Du B.A. (2006). Selective determination of cysteine by resonance light scattering technique based on self-assembly of gold nanoparticles. Anal. Biochem..

[24] Wu Y.-Q., Xiao H.-S., Peng Y.-Y., Wu B.-W., Li B., Ding Y.-C., Li D., Wu L., Yu D., Cao Z. (2024). Simple, rapid and enzyme-free assay for potentiometric determination of L-cysteine based on *meso*-2,3-dimercaptosuccinic acid self-assembled gold electrode. Microchem. J..

[25] Peng Y.-Y., Wang Y., Yu X., Zeng J., Xiao Z.-L., Cao Z. (2020). Rapid and sensitive detection of *L*-cysteine based on mono(6-mercapto-6-deoxy)-*β*-cyclodextrin modified gold electrode. Chem. J. Chin. Univ..

[26] Cao Z., Li W.-F., Liu C., Peng Y.-Y., Huang Y., Xiao Z.-L. (2021). Design and fabrication of embedded wireless monitoring system based on Bluetooth low energy transmission for potentiometric sensors. Chin. J. Anal. Chem..

[27] Huang L., Li J., Wang Y., Hu X., Shi K., Li C., Xie Y., Zhao P., Fei J. (2023). A light-driven enzyme-free photoelectrochemical sensor based on HKUST-1 derived Cu_2_O/Cu@microporous carbon with g-C_3_N_4_ p-n heterojunction for ultra-sensitive detection of L-cysteine. Carbon.

[28] Wu L., Zhu K., Xue S., Wu B., Xiao Z., Feng Z., Yin Y., Li J., Yu D., Cao Z. (2024). Dual-mode arginine assay based on the conformation switch of a ferrocene-grafted polypeptide. Anal. Chem..

[29] Wang W., He Y., He S., Liu X., Gui Q.-W., Deng L., Wang H., Cao Z., Feng Z., Xiong B. (2024). Peptide aptamer-based colorimetric sensor for the detection of L-tryptophan in porcine serum. Microchem. J..

[30] Chen Z., Li S., Guan Y., Wu C., Qian Y., Zhou H., Qian Y., Yue Y., Yue W. (2024). Ultrasmall CuMn-His nanozymes with multienzyme activity at neutral pH: Construction of a colorimetric sensing array for biothiol detection and disease identification. ACS Appl. Mater. Interfaces.

[31] Wu Y.-Y., Tian X., Jiang Y., Ma H.-Y., Wang W., Zhang W.-S., Martin J.S., Yan Y., Qin D.-D., Han D.-X. (2024). Advances in bimetallic materials and bimetallic oxide nanozymes: Synthesis, classification, catalytic mechanism and application in analytical chemistry. TrAC Trends Anal. Chem..

[32] Wan K., Jiang B., Tan T., Wang H., Liang M. (2022). Surface-mediated production of complexed •OH radicals and Fe=O species as a mechanism for iron oxide peroxidase-like nanozymes. Small.

[33] Gao L., Zhuang J., Nie L., Zhang J., Zhang Y., Gu N., Wang T., Feng J., Yang D., Perrett S. (2007). Intrinsic peroxidase-like activity of ferromagnetic nanoparticles. Nat. Nanotechnol..

[34] Zhang Q., Li M., Guo C., Jia Z., Wan G., Wang S., Min D. (2019). Fe_3_O_4_ nanoparticles loaded on lignin nanoparticles applied as a peroxidase mimic for the sensitively colorimetric detection of H_2_O_2_. Nanomaterials.

[35] Ahmed S.R., Cirone J., Chen A. (2019). Fluorescent Fe_3_O_4_ quantum dots for H_2_O_2_ detection. ACS Appl. Nano Mater..

[36] Su L., Qin W., Zhang H., Rahman Z.U., Ren C., Ma S., Chen X. (2015). The peroxidase/catalase-like activities of MFe_2_O_4_ (M=Mg, Ni, Cu) MNPs and their application in colorimetric biosensing of glucose. Biosens. Bioelectron..

[37] Zou W., Tang Y., Zeng H., Wang C., Wu Y. (2021). Porous Co_3_O_4_ nanodisks as robust peroxidase mimetics in an ultrasensitive colorimetric sensor for the rapid detection of multiple heavy metal residues in environmental water samples. J. Hazard. Mater..

[38] Alizadeh N., Salimi A., Hallaj R. (2019). Mimicking peroxidase-like activity of Co_3_O_4_-CeO_2_ nanosheets integrated paper-based analytical devices for detection of glucose with smartphone. Sens. Actuators B Chem..

[39] Vinothkumar G., Lalitha A.I., Suresh Babu K. (2019). Cerium phosphate–cerium oxide heterogeneous composite nanozymes with enhanced peroxidase-like biomimetic activity for glucose and hydrogen peroxide sensing. Inorg. Chem..

[40] Wang L., Chu Y., Cao B., Zhang R., Hussain Z., Liu Q. (2025). Cobalt (II) porphyrin nanoaggregates as sacrificial templates to improve the peroxidase-like activity of light-controlled TiO_2_-based nanozymes for colorimetric determination of amikacin. Talanta.

[41] Zhang W., Lan Y., Chai D.-F., Lv J., Dong G., Guo D. (2024). A novel “On-Off” colorimetric sensor for ascorbic acid and hydrogen peroxide based on peroxidase activity of CeO_2_/Co_3_O_4_ hollow nanocubes. J. Mol. Struct..

[42] Xu Y., Zhou J., Sun P., Li P., Han D., Wang X., Chai F. (2025). The construction of peroxidase-like Cu-BDC@FeMo_6_ for colorimetric detection of H_2_O_2_ and dopamine. Sep. Purif. Technol..

[43] Lei M., Ding X., Liu J., Tang Y., Chen H., Zhou Y., Zhu C., Yan H. (2024). Trace amount of Bi-doped core–shell Pd@Pt mesoporous nanospheres with specifically enhanced peroxidase-like activity enable sensitive and accurate detection of acetylcholinesterase and organophosphorus nerve agents. Anal. Chem..

[44] Yang Q.-Y., Wan C.-Q., Wang Y.-X., Shen X.-F., Pang Y.-H. (2023). Bismuth-based metal-organic framework peroxidase-mimic nanozyme: Preparation and mechanism for colorimetric-converted ultra-trace electrochemical sensing of chromium ion. J. Hazard. Mater..

[45] Yu K., Li M., Chai H., Liu Q., Hai X., Tian M., Qu L., Xu T., Zhang G., Zhang X. (2023). MOF-818 nanozyme-based colorimetric and electrochemical dual-mode smartphone sensing platform for in situ detection of H_2_O_2_ and H_2_S released from living cells. Chem. Eng. J..

[46] Wang X., Han Q., Cai S., Wang T., Qi C., Yang R., Wang C. (2017). Excellent peroxidase mimicking property of CuO/Pt nanocomposites and their application as an ascorbic acid sensor. Analyst.

[47] Wu L.-L., Wang L.-Y., Xie Z.-J., Pan N., Peng C.-F. (2016). Colorimetric assay of L-cysteine based on peroxidase-mimicking DNA-Ag/Pt nanoclusters. Sens. Actuators B Chem..

[48] Li X., Yang X., Cheng X., Zhao Y., Luo W., Elzatahry A.A., Alghamdi A., He X., Su J., Deng Y. (2020). Highly dispersed Pt nanoparticles on ultrasmall EMT zeolite: A peroxidase-mimic nanoenzyme for detection of H_2_O_2_ or glucose. J. Colloid Interface Sci..

[49] Cheng Y., Shen P., Li X., Li X., Chu K., Guo Y. (2023). Synergistically enhanced peroxidase-like activity of Fe_3_O_4_/Ti_3_C_2_ MXene quantum dots and its application in colorimetric determination of Cr (VI). Sens. Actuators B Chem..

[50] Peng L.-J., Zhang C.-Y., Zhang W.-Y., Zhou H.-Y., Yang F.-Q. (2022). The peroxidase-like catalytic activity of in situ prepared cobalt carbonate and its applications in colorimetric detection of hydrogen peroxide, glucose and ascorbic acid. Colloids Surf. A Physicochem. Eng. Asp..

[51] Zandieh M., Liu J. (2023). Nanozymes: Definition, activity, and mechanisms. Adv. Mater..

[52] Shen X., Wang Z., Gao X.J., Gao X. (2023). Reaction mechanisms and kinetics of nanozymes: Insights from theory and computation. Adv. Mater..

[53] Khramtsov P., Minin A., Galaeva Z., Mukhlynina E., Kropaneva M., Rayev M. (2023). Optimizing the composition of the substrate enhances the performance of peroxidase-like nanozymes in colorimetric assays: A case study of prussian blue and 3,3′-diaminobenzidine. Molecules.

[54] Bauduin P., Nohmie F., Touraud D., Neueder R., Kunz W., Ninham B.W. (2006). Hofmeister specific-ion effects on enzyme activity and buffer pH: Horseradish peroxidase in citrate buffer. J. Mol. Liq..

[55] Chen S., Chi M., Zhu Y., Gao M., Wang C., Lu X. (2018). A Facile synthesis of superparamagnetic Fe_3_O_4_ nanofibers with superior peroxidase-like catalytic activity for sensitive colorimetric detection of l-cysteine. Appl. Surf. Sci..

[56] Zhao H., Liu K., Zhou L., Zhang T., Han Z., Wang L., Ji X., Cui Y., Hu J., Ma G. (2023). Platinum palladium bimetallic nanozymes stabilized with vancomycin for the sensitive colorimetric determination of L-cysteine. Biomolecules.

[57] Lin X., Wang C., You L., Fu F., Liu Q. (2023). Nanozyme colorimetric sensing of L-cysteine and copper ions based on PtCo nanoparticles@multi-walled carbon nanotubes. Anal. Sci..

[58] Kavitha S., Mary Jelastin Kala S., Anand Babu Christus A., Ravikumar A. (2021). Colorimetric determination of cysteine and copper based on the peroxidase-like activity of Prussian blue nanocubes. RSC Adv..

[59] Wei Z., Yang L., Ou M., Xie Y., Zhao C. (2024). Tailoring the enzyme-mimetic activity and surface nanostructure of metal-phenolic platform for colorimetric detection of L-cysteine. ACS Sustain. Chem. Eng..

[60] Xu D., Tu Q., San X., Zhu A., Li X. (2024). CoO/Co-graphene quantum dots as an oxidative mimetic nanozyme for the colorimetric detection of L-cysteine. Anal. Methods.

[61] Jia W.-X., Tao M.-M., Wang X.-Y., Huang Y.-F., Li Y. (2024). Bimetallic MOFs-derived four-in-one CeCuOx/C nanozyme and its application for colorimetric detection of cysteine based on oxidase mimics. Talanta Open.

[62] Wang W., Ge X., Chen D., Wu L., Ge D., Chen X. (2024). Co nanoparticles confined in N-doped hollow carbon nanospheres as multifunctional oxidase mimic for colorimetric detection of L-cysteine and Hg^2+^. J. Environ. Chem. Eng..

[63] Alula M.T. (2023). Peroxidase-like activity of biosynthesized silver nanoparticles for colorimetric detection of cysteine. RSC Adv..

[64] Tian L., Cheng C., Zhao Z., Liu W., Qi L. (2023). Enhancing the catalytic performance of MOF-polymer@AuNP-based nanozymes for colorimetric detection of serum L-cysteine. Analyst.

[65] Yi Z., Ren Y., Li Y., Li Y., Long F., Zhu A. (2023). Rational design of three-in-one Pt/MnO_2_/GO hybrid nanozyme with enhanced catalytic performance for colorimetric detection of ascorbic acid and cysteine. Talanta.

[66] Huang N., Yang D., Chen H., Xiao Y., Wen J., Long Y., Zheng H. (2023). Colorimetric detection of biothiols and Hg^2+^ based on the peroxidase-like activity of GTP. Spectrochim. Acta Part A Mol. Biomol. Spectrosc..

[67] Feng S., Wen F., He L., Su J., Jiang P., He D. (2022). Facile synthesis of ultrathin MnO_2_ nanobelts anchoring on porous carbon with high oxidase mimetic activity for L-cysteine colorimetric detection. Sens. Actuators B Chem..

[68] Ponnaiah S.K., Periakaruppan P., Vellaichamy B. (2018). New electrochemical sensor based on a silver-doped iron oxide nanocomposite coupled with polyaniline and its sensing application for picomolar-level detection of uric acid in human blood and urine samples. J. Phys. Chem. B.

