# Supplementary material for: Pt@ZnCo2O4 Microspheres as Peroxidase Mimics: Enhanced Catalytic Activity and Application for L-Cysteine Detection
Source: Molecules. 2025 Jan 5;30(1):187. doi: 10.3390/molecules30010187 (PMC11722260; doi:10.3390/molecules30010187)
Supplement: Supplementary file 1 [file molecules-30-00187-s001.zip › molecules-3347437-supplementary.pdf]

Supporting Information

Pt@$ZnCo_2O_4$ Microspheres as Peroxidase Mimics: Enhanced Catalytic Activity and Application for L-Cysteine Detection

Shuqi Liao[1,†], Weisen Deng[1,†], Feng Yang[1], Jutao Zhou[1], Ling Wu[1], Donghong Yu[2], Zhong Cao[1,*]

[1] Hunan Provincial Key Laboratory of Materials Protection for Electric Power and Transportation & Hunan Provincial Key Laboratory of Cytochemistry, School of Chemistry and Chemical Engineering, Changsha University of Science and Technology, Changsha 410114, China; liaoshuqi@stu.csust.edu.cn (S.L.), senweideng@163.com (W.D.), yangf2004@stu.csust.edu.cn (F.Y.), zhoujutao@stu.csust.edu.cn (J.Z.), wuling@csust.edu.cn (L.W.), caoz@csust.edu.cn (Z.C.)

[2] Department of Chemistry and Bioscience, Aalborg University, DK-9220 Aalborg, East, Denmark; yu@bio.aau.dk (D.Y.)

----

* Corresponding author. E-mail: caoz@csust.edu.cn (Z.C.)

† S.L. and W.D. contributed equally to this work as co-first authors.

Figure S1 3

Figure S2 4

Figure S3 5

Figure S4 6

Figure S5 7

Table S1 8

Table S2 9

Table S3 10

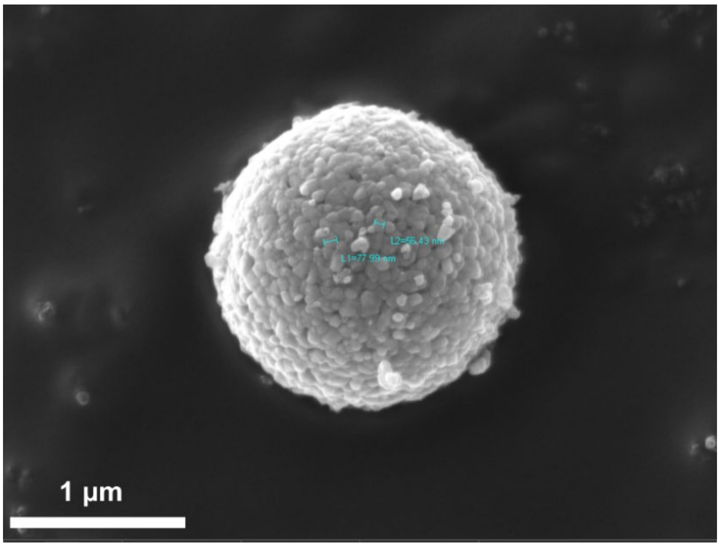

Figure S1. Particle size distribution of $ZnCo_2O_4$ microspheres formed by aggregation.

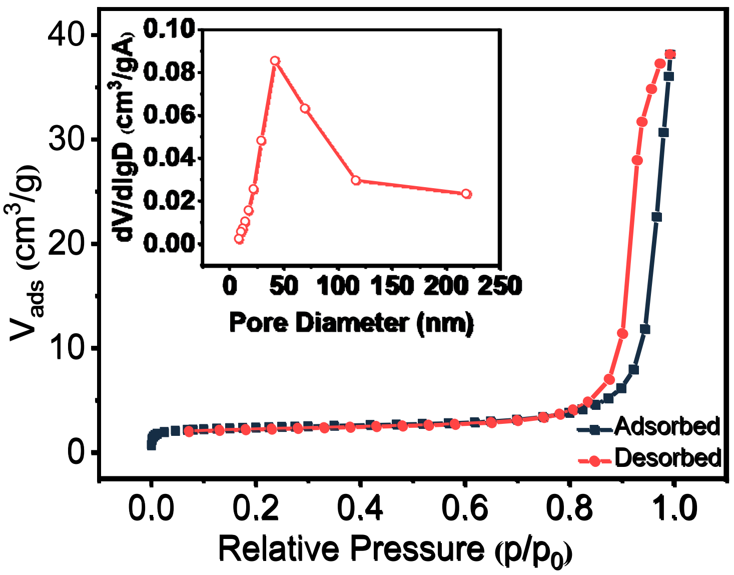

Figure S2. N_2 adsorption−desorption isotherm and pore size distribution diagram of $ZnCo_2O_4$.

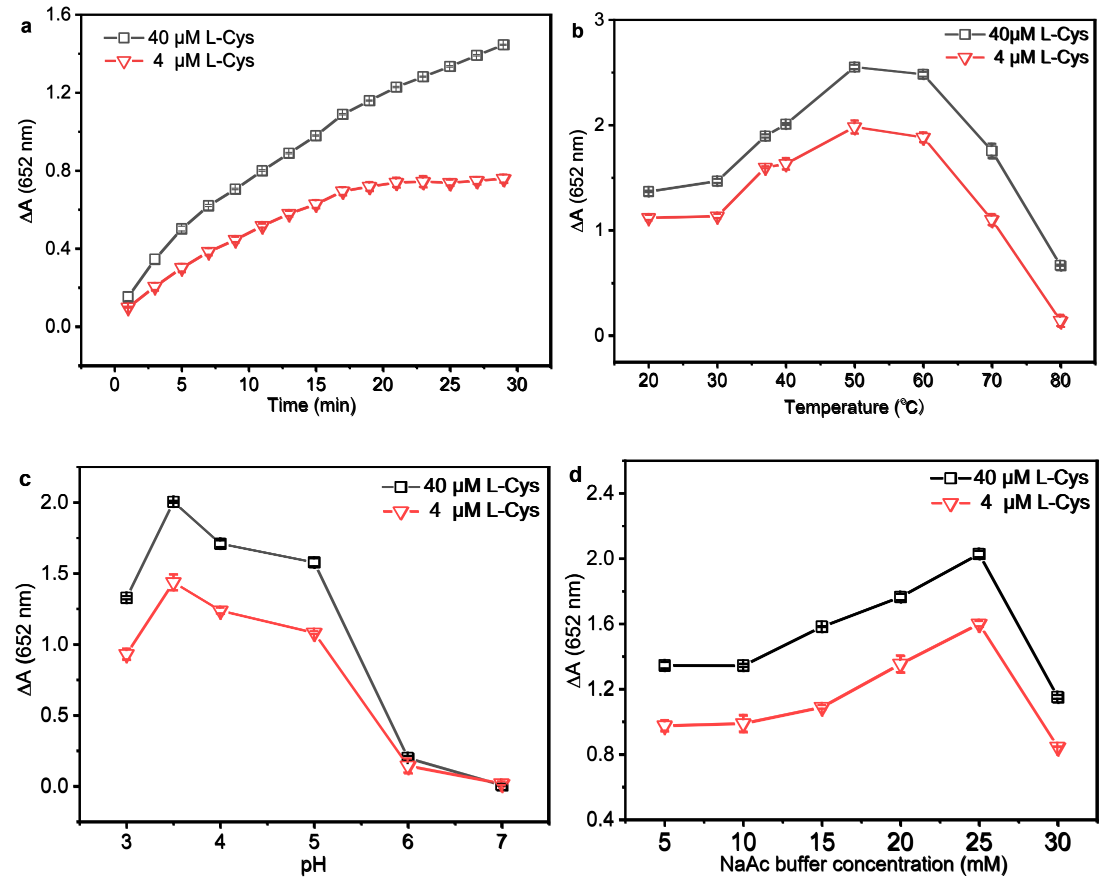

Figure S3. Effects of reaction time (a), temperature (b), pH (c), and NaAc concentration (d) on the colorimetric sensor for detecting L-Cys.

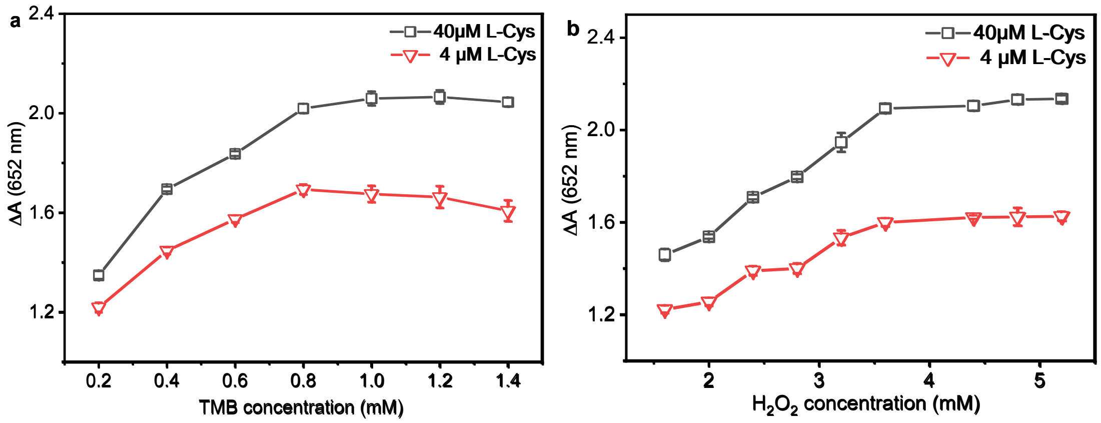

Figure S4. Effects of TMB (a) and H_2O_2 concentrations on the detection of L-Cys using the colorimetric sensor.

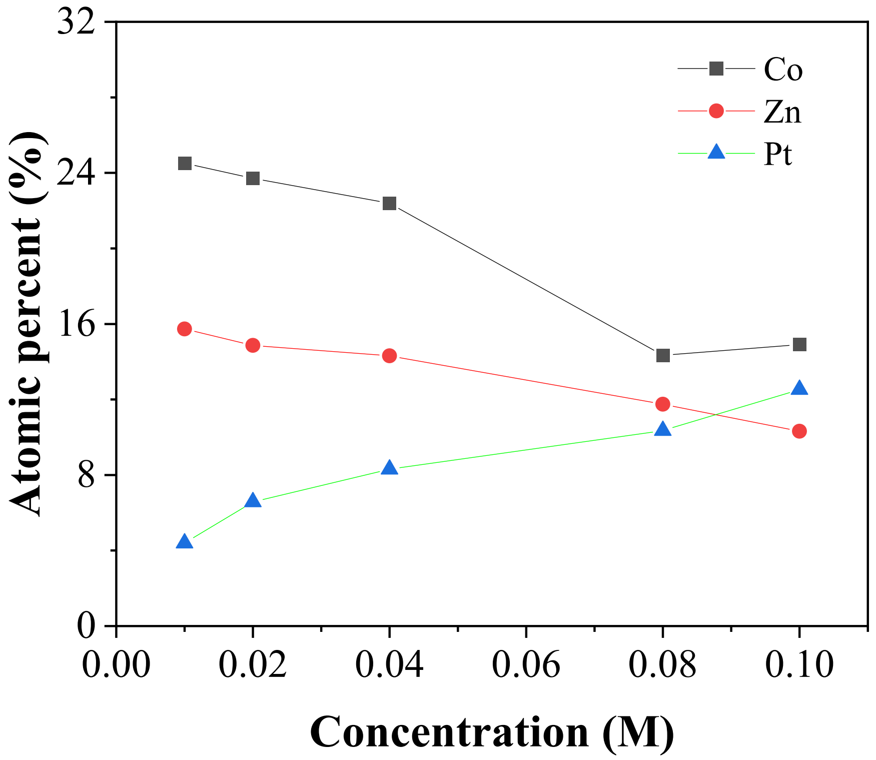

Figure S5. Atomic percentages of Co, Zn, and Pt in 4.39% Pt@$ZnCo_2O_4$, 6.58% Pt@$ZnCo_2O_4$, 8.31% Pt@$ZnCo_2O_4$, 10.4% Pt@$ZnCo_2O_4$, and 12.5% Pt@$ZnCo_2O_4$ with corresponding concentrations of chloroplatinic acid solutions added (0.01 M, 0.02 M, 0.04 M, 0.08 M, and 0.10 M).

Table S1. Comparison of K_m and V_{max} values between $ZnCo_2O_4$ microspheres and 12.5% Pt@$ZnCo_2O_4$ microspheres.

Catalyst	K_m (mM)		V_{max} ($10^{-8}MS^{-1}$)	
	TMB	H_2O_2	TMB	H_2O_2
$ZnCo_2O_4$	0.387	3.19	5.66	6.62
12.5% Pt@$ZnCo_2O_4$	0.112	1.32	21.1	21.4

Table S2. Contents of metal elements in Pt@$ZnCo_2O_4$ microspheres at varying levels of Pt loaded.

Sample [a]	Pt (At%) [b]	Zn (At%) [b]	Co (At%) [b]
Pt@$ZnCo_2O_4$ (0.01M)	4.39	15.73	24.51
Pt@$ZnCo_2O_4$ (0.02M)	6.58	14.86	23.71
Pt@$ZnCo_2O_4$ (0.04M)	8.31	14.31	22.39
Pt@$ZnCo_2O_4$ (0.08M)	10.4	11.75	14.34
Pt@$ZnCo_2O_4$ (0.10M)	12.5	10.32	14.91

Note: [a] Pt@$ZnCo_2O_4$ MSs were synthesized by using different concentrations of chloroplatinic acid solutions (0.01 M, 0.02 M, 0.04 M, 0.08 M, and 0.10 M). [b] Corresponding atomic percentages of metal elements were obtained from the elemental distribution analysis.

Table S3. Active surface area of different modified electrodes.

Modified electrode	Oxidation peak current (A)	Active surface area (cm^2)
Bare GCE	3.607×10^{-6}	1.283×10^{-3}
$ZnCo_2O_4$/GCE	3.683×10^{-6}	1.310×10^{-3}
Pt@$ZnCo_2O_4$/GCE	8.358×10^{-6}	2.973×10^{-3}

Note: The different modified electrodes were examined in a TMB (0.4 mM) containing 25 mM sodium acetate-acetic acid buffer (pH 3.5) solution. Their corresponding active surface areas were evaluated by using the Randles−Sevcik equation [68].

$$I_p = 2.69\times10^5 n^{3/2} A D^{1/2} C v^{1/2}$$

where D is the diffusion coefficient of TMB ($cm^2\ s^{-1}$), I_p is the anodic peak current (A), C is the concentration of TMB ($mol\ cm^{-3}$), A is the electro active area (cm^2), and n is the number of transferred electrons.

References

68. Ponnaiah, S. K.; Periakaruppan, P.; Vellaichamy, B. New electrochemical sensor based on a silver-doped iron oxide nanocomposite coupled with polyaniline and its sensing application for picomolar-level detection of uric acid in human blood and urine samples. *J. Phys. Chem. B* **2018,** *122*, 3037-3046.
